# Physical exercise in facioscapulohumeral muscular dystrophy: state of the art and future challenges beyond common misconceptions

**DOI:** 10.1007/s00421-026-06338-y

**Published:** 2026-07-02

**Authors:** Oscar Crisafulli, Matteo Fortunati, Fabrizio Brindisi, Andi Nuredini, Stefano Palermi, Rossella Tupler, Nicoline Voet, Anna Odone, Giuseppe D’Antona

**Affiliations:** 1 Centro di Ricerca Interdipartimentale Attività Motorie e Sportive (CRIAMS) Sport Medicine Centre Voghera, University of Pavia, Voghera, 27058 Italy; 2 Department of Industrial Engineering, University of Tor Vergata, Rome, 00133 Italy; 3 Facultad de Deportes, Universidad Católica San Antonio de Murcia, Guadalupe, 30107 Spain; 4 Department of Biomedical, Metabolic and Neural Sciences, University of Modena and Reggio Emilia, Modena, Italy; 5 Department of Medicine and Surgery, UniCamillus Saint Camillus International University of Health Sciences, Rome, 00187 Italy; 6 Department of Rehabilitation, Donders Institute for Brain, Cognition and Behaviour, Radboud University Medical Center, Nijmegen, The Netherlands; 7 Klimmendaal, Rehabilitation Center, Arnhem, The Netherlands; 8 Department of Public Health, Experimental and Forensic Medicine, University of Pavia, Pavia, 27100 Italy; 9 Medical Direction, Fondazione IRCCS Policlinico San Matteo, Pavia, Italy

**Keywords:** Facioscapulohumeral muscular dystrophy, Neuromuscular disease, Non-pharmacological intervention, Physical exercise, Aerobic exercise, Anaerobic exercise, Nutritional strategies

## Abstract

Facioscapulohumeral muscular dystrophy (FSHD) is a progressive inherited myopathy characterized by muscle weakness, fatty infiltration, and consequent reduction in functional capacity. In this context, physical exercise (PE) has been proposed as a non-pharmacological strategy aimed at preserving physical efficiency. However, its prescription in FSHD has historically been approached with caution due to concerns regarding overwork weakness and potential exacerbation of muscle damage. As a result, clinical management has often remained conservative, with limited and non-standardized PE recommendations. Nevertheless, physical inactivity may further contribute to deconditioning, loss of autonomy, and deterioration in quality of life, highlighting a clear tension between perceived risk and potential benefit. In this framework, identifying safe and effective PE strategies represents a central unmet need in FSHD care. Possibly due to disease rarity and historical concerns, the available literature on PE in FSHD remains fragmented, limiting the strength of definitive conclusions. A structured synthesis of existing evidence may help clarify feasible and potentially effective exercise modalities, as well as guide the development of optimized intervention strategies. This review critically examines the current evidence on PE in FSHD, with specific reference to aerobic, anaerobic, and combined training interventions. The potential synergistic role of concomitant nutritional strategies is also considered. The critical analysis highlights exercise modalities that appear more consistently safe and potentially beneficial, while underscoring substantial methodological limitations across studies. Finally, emerging concepts in exercise tailoring are discussed, with the aim of addressing the clinical variability of FSHD and informing future prospective, controlled investigations.

**Supplementary Information:**

The online version contains supplementary material available at https://doi.org/10.1007/s00421-026-06338-y.

## Introduction

Facioscapulohumeral muscular dystrophy (FSHD) is one of the most prevalent inherited myopathies, with an estimated frequency of approximately 1 in 15,000–20,000 individuals (Flanigan et al. 2001). The disorder is characterized by progressive, often asymmetric muscle degeneration and fatty infiltration, primarily affecting the facial, scapular, and upper-limb regions, with subsequent involvement of axial and lower-limb muscles in a substantial proportion of patients (Schätzl et al. 2021). Consequently, many individuals experience reduced physical efficiency, limitations in daily activities, and varying degrees of disability over time (Eichinger et al. 2018). In the absence of therapies, current management strategies focus on preserving functional capacity and preventing secondary complications, with physical exercise (PE) increasingly recognized as a potentially valuable non-pharmacological intervention.

However, the implementation of PE in FSHD has historically been controversial. Similar to other dystrophic conditions (Fowler 1984; Fowler and Taylor 1982), the primary concern has been the potential development of overwork weakness syndrome, defined as an irreversible loss of muscle strength resulting from excessive loading of already compromised muscle (Johnson and Braddom 1971). Consequently, it has been suggested that even routine activities of daily living may already impose a substantial muscular workload on patients (McDonald 2010), raising the possibility that additional PE could be excessive or potentially harmful. In this context, additional apprehension has arisen from evidence that high-intensity PE can acutely elevate inflammatory markers (Cerqueira et al. 2019). This is particularly relevant because counteracting inflammation has been identified as an important clinical goal (Ansari et al. 2025), given its established link with muscle degeneration in FSHD (Dahlqvist et al. 2020; Pini et al. 2026). In fact, although direct evidence that exercise-induced acute inflammation causes muscle degeneration is lacking, this concern has occasionally discouraged clinicians from recommending any form of PE, partly because objective and reliable criteria for defining what constitutes high-intensity activity for a compromised muscle were, and still are, lacking.

Although growing evidence indicates that aerobic exercise (Olsen et al. 2005; Voet et al. 2014) is both feasible and beneficial in FSHD, the most appropriate and beneficial PE modalities for FSHD remain a topic of ongoing discussion (Ansari et al. 2025; Gianola et al. 2020). In particular, due to the rarity of the condition (Bettio et al. 2021), the current literature is fragmented and largely based on small patient cohorts, which offer limited inferential value, particularly given the wide variability in clinical manifestations (Ricci et al. 2016). Furthermore, studies on higher-intensity or predominantly anaerobic approaches, although available (Andersen et al. 2017; Bankolé et al. 2016), remain comparatively scarce. This results in substantial gaps regarding modality-specific safety, exercise-induced adaptations and clinical relevance.

This fragmentation also extends to studies examining the combined effects of PE and nutritional interventions. It is well established that both aerobic and anaerobic training adaptations can be enhanced through the synergistic effects of appropriate nutritional strategies and/or supplementation (Deng et al. 2025; Naderi et al. 2025). However, evidence in the context of FSHD remains limited and inconsistent, with only a few studies conducted in small patient cohorts (Andersen et al. 2015) leading to a lack of consensus.

In this context, synthesizing the available literature within a unified conceptual framework is essential to evaluate the feasibility, safety, and efficacy of PE in FSHD, to outline emerging models for sustainable and condition-specific exercise prescription, and to identify key physiological, methodological, and clinical gaps that should guide future research. Accordingly, this review critically examines PE in individuals with FSHD, explicitly distinguishing between aerobic and anaerobic training modalities and considering studies that combine exercise interventions with nutritional strategies.

## Methods

This review focused on English-language articles investigating PE interventions in individuals diagnosed with FSHD. Relevant literature was identified through searches of PubMed and Web of Science from inception to December 2025, using a combination of keywords and major subject headings, including “physical exercise”, “physical training”, “facioscapulohumeral dystrophy”, “aerobic training”, “anaerobic training”, “nutrition”, and “supplementation”. It is important to note that this review did not use a systematic search strategy; therefore, some relevant literature may have been overlooked. Given the limited number of available studies, the marked heterogeneity in interventions, outcome measures, and study designs, and the exploratory aim of providing a clinically oriented overview of exercise interventions in FSHD, a narrative approach was considered more appropriate than a formal systematic review. Eligibility criteria included studies reporting pre- and post-training assessments of functional, physiological, or clinical outcomes. Studies reporting only single exercise sessions were excluded, along with studies combining exercise with pharmacological treatments, studies including mixed neuromuscular populations without separate FSHD analyses, and conference papers. Both randomized controlled trials (RCTs) and non-randomized studies were considered. Case reports were also included to provide a more comprehensive overview of the available data. To facilitate interpretation, studies were stratified into three categories based on study design and presented in separate sections. Within each section, evidence was further organized into additional subsections: aerobic (AET), anaerobic (ANET), and combined exercise training. Finally, studies including concomitant nutritional interventions were classified according to the same criteria. The aim of the study descriptions is to provide a comprehensive overview of patient characteristics, study objectives, outcome assessment methodologies, training protocols and results. To enhance clarity and usability, the results are reported only as the direction of observed changes and, for group studies, statistical significance. Numerical details are instead summarized in supplementary material. In the Discussion, we critically evaluated the available evidence, emphasizing safe and effective interventions, reporting any exercise-induced adverse outcomes, highlighting research gaps, and proposing future research objectives for the development of tailored exercise strategies in FSHD.

### Randomized controlled trials

#### Aerobic exercise training

Two randomised controlled trials (RCTs) have investigated the effects of aerobic exercise training (AET) in patients with FSHD (Janssen et al. 2016; Voet et al. 2014).

In the trial of Voet et al. (2014), 57 patients with FSHD type 1 were randomised to an AET group (*n* = 20; median age 59 years, eight females, median Ricci Score 3.0), a cognitive-behavioural training (CBT) group (*n* = 13; median age 49 years, five females, median Ricci Score 2.0) or a usual care (UC) group (*n* = 24; median age 52 years, seven females, median Ricci Score 3.0). The research design comprised an initial 28-week parallel-group phase (16-week intervention plus 12-week follow-up), followed by a second phase in which the UC group was re-randomised to receive either AET or CBT, plus a further 12-week follow-up period. This design ensured a comparable duration of usual care across all groups before the follow-up assessment.

The primary aim of the study was to evaluate the effects of AET and CBT on chronic fatigue, as measured by the Checklist Individual Strength fatigue subscale (CIS-fatigue) questionnaire. Secondary aims were to evaluate the effects on muscle strength, aerobic exercise tolerance, physical endurance, and daily activity levels. Muscle strength was measured as the maximal voluntary isometric contraction (MVIC) of the quadriceps using a quantitative muscle testing system. Aerobic exercise tolerance was estimated using the Astrand test and measured as peak oxygen uptake (V̇O₂peak). Physical endurance was measured by the distance travelled during the 6-minute walking test (6MWT), and daily activity was monitored by body accelerations collected using an ankle actometer worn for 12 consecutive days. These evaluations were performed at pre-intervention, post-intervention (+ 16 weeks) and follow-up (+ 12 additional weeks, resulting in + 28 weeks).

The AET intervention consisted of cycle ergometer exercise performed three times per week, with session duration of 30 min and intensity progressively increased from 50% to 65% of heart rate reserve (HRR). CBT consisted of 50-minute sessions targeting maladaptive coping strategies, whereas the UC group received no structured intervention or only occasional conventional physiotherapy.

Between-group analyses at post-intervention (+ 16 weeks) demonstrated that AET, compared to UC, significantly reduced CIS-fatigue, while significantly augmenting MVIC and daily activity. The same results also applied when comparing CBT and UC results, except for a non-significant between-group change in muscle strength. However, at follow-up (+ 28 weeks) AET and CBT remained significant compared with UC just in CIS-fatigue and daily activities. Of note, the absence of within-group analyses limits the interpretation of the direction and magnitude of changes within each intervention arm. Moreover, no comparison between AET and CBT was performed, limiting the ability to determine the relative efficacy of the two interventions.

The effects of AET and CBT on muscle composition were the focus of a subsequent study by Janssen et al. (2016), which built on the findings of earlier research. In this investigation, adopting the same research design as described in Voet et al. (2014), a total of 31 patients were included as follows: AET (*n* = 9; mean age 56 ± 15 years, 4 females, Clinical Severity Score (CSS) 3.1 ± 1.1), CBT (*n* = 9; mean age 53 ± 12 years, 3 females, CSS 2.3 ± 1.0), and UC (*n* = 13; mean age 53 ± 15 years, 3 females, CSS 3.0 ± 0.9). The AET and CBT interventions were consistent with those described by Voet et al. (2014). As in the previous study, the UC group received standard care, which typically consisted of no treatment or only occasional conventional physiotherapy.

The outcome was the progression of fatty infiltration in thigh muscles, assessed using quantitative magnetic resonance imaging (MRI) at baseline and follow-up. Notably, in the AET and CBT groups, MRI acquisition was performed after the intervention period, followed by an additional 12 weeks of usual care, whereas in the UC group, it was conducted only after 12 weeks. The findings indicated that both AET and CBT reduced significantly the velocity of progression of fatty infiltration in thigh muscles compared to UC.

#### Anaerobic exercise training

To date, only one RCT has examined the feasibility and effects of high-intensity interval training (HIT) in patients with FSHD type 1 (Andersen et al. 2017). Of note, HIT protocols are generally considered a predominantly, although not exclusively, anaerobic exercise training (ANET) modality because they rely on repeated short bouts of high-intensity effort interspersed with recovery periods (Buchheit and Laursen 2013). Importantly, HIT also places substantial demands on aerobic metabolism and is well recognized to induce aerobic adaptations (MacInnis and Gibala 2017).

In this study, 12 patients were randomised to either a supervised HIT group (*n* = 6; mean age 53 ± 15 years, 2 females, FSHD Clinical Score: 6.3 ± 4.3) or a UC group (*n* = 6; mean age 46 ± 9 years, 1 female, FSHD Clinical Score: 7.5 ± 2.1), while an additional group of age- and sex-matched healthy controls (HC) performed the same training protocol. The intervention consisted of two consecutive phases: during the first 8 weeks, patients were randomised to supervised HIT or UC, while in the subsequent 8 weeks, all FSHD patients performed unsupervised HIT. Similarly, the group of HC performed the same supervised (first 8 weeks) and unsupervised (9th to 16th weeks) HIT protocol.

Outcomes, evaluated at pre- and post-intervention, were changes in maximal oxygen uptake (V̇O₂max) and maximal workload (Wmax), during a cycle-ergometer cardiopulmonary exercise test (CPET), along with 6MWT, 5-time sit-to-stand-test (5STS), isometric muscle strength of hip flexion, knee extension and flexion, and elbow flexion measured with a handheld dynamometer, and daily activity levels using a pedometer. Moreover, for safety reasons, plasma creatin kinase (CK) was evaluated at baseline and during the first phase of the HIT protocol. However, the temporal schedule for plasma CK assessment was not specified.

As the first study implementing HIT in patients with FSHD, the intervention was based on a protocol previously shown to improve V̇O₂max and running performance in moderately trained healthy individuals (Gunnarsson and Bangsbo 2012). The programme consisted of three weekly sessions, each lasting 21 min and comprising repeated short bouts of alternating low, moderate, and maximal intensities. Each session included two 5-minute blocks separated by a 3-minute recovery period at very low intensity. Within each minute, exercise intensity alternated between 30 s of low-intensity pedalling, 20 s of moderate to high intensity, and 10 s of maximal effort. The overall session intensity corresponded to approximately 80 ± 7% of maximum heart rate (HRmax) in patients with FSHD and 78 ± 4% in HC. The activities performed by the UC group were not further specified.

Within-group comparisons showed that 8 weeks of HIT led to significant improvements in V̇O₂max and Wmax in the HIT group. Conversely, the UC group had no improvements in either V̇O₂max and Wmax, while the HC group performing HIT only had a significant improvement in Wmax. Regarding isometric muscle strength, 5STS, and daily activity levels, no group had a significant change from baseline, while in the 6MWT, only the UC group had an improvement. During the additional unsupervised HIT period, the FSHD HIT group maintained its V̇O₂max and Wmax compared to the first training period without additional gains, while the FSHD UC group had a significant improvement in both outcomes compared to baseline. Whereas in HC, there was no further significant improvement of V̇O₂max and Wmax compared to baseline values. Additionally, the authors reported no episodes of muscle damage, stable CK plasma level during the HIT protocol compared with baseline values, as well as the self-rated levels of muscle pain.

#### Combined aerobic and anaerobic exercise training

Two studies, both referring to the same cohort, have investigated the effects of a combined aerobic and strength training program in patients with FSHD type 1 (Bankolé et al. 2016; Horwath et al. 2025). This approach was tested considering previous evidence in patients with neuromuscular disorders suggesting that it may be more effective than aerobic exercise training alone (Cup et al. 2007; Voet 2019).

The study by Bankolé et al. (2016) involved a trial divided in two phases. In the first phase, 16 patients were randomised into a training group (TG) (*n* = 8; mean age 40 ± 13 years; 3 females) or a control group (CG) (*n* = 8; mean age 41 ± 9 years; 1 female). In the second phase, all CG participants underwent the same training programme.

Specifically, the program consisted of a combined aerobic and strength exercise using a home-based stationary cycle ergometer for 24 weeks, with a training frequency of three sessions per week and a duration of 35 min per session. Two of the weekly sessions consisted of a combined protocol including a 5-minute warm-up followed by 20 min of aerobic training performed at a constant moderate intensity corresponding to 60% of maximal aerobic power (MAP). This was followed by a brief strength-oriented component consisting of five sets of 10 near-maximal pedal revolutions completed within 5 min. The third weekly session consisted of five 1-minute intervals performed at 80% of MAP, each interspersed by 5 min of active recovery at 40% MAP, plus warm-up and cool-down phases, still at 40% MAP.

Outcomes included V̇O₂peak, muscle histology, functional performance (strength, fatigue, endurance), and quality of life. V̇O_2_peak and MAP were measured during a CPET on a cycler-ergometer, while functions were evaluated through the 6MWT, MVIC of the quadriceps with femoral nerve magnetic stimuli delivered during the isometric contraction and at rest, and through the number of repetitions until task failure. Moreover, the quality of life and perceived fatigue were assessed with the Short Form Health Survey (SF-36) and Fatigue Severity Scale (FSS). Muscle histological characteristics were evaluated as muscle cross-sectional area (CSA) and enzyme activities through biopsies from the vastus lateralis. All the procedures were performed at pre- and post-intervention. Additionally, serum CK concentrations, as a marker of muscle damage, were assessed 24 h after neuromuscular and physiological testing, resulting in five time points, namely at baseline and every 6 weeks.

The aerobic and strength-oriented training significantly improved all the outcomes investigated in the TG (i.e. significantly increasing V̇O_2_peak, MAP, MVIC, number of repetitions. 6MWT, muscle fibre CSA, and Citrate synthase activity, while significantly reducing FSS), except for a non-statistically changing in the SF-36. On the contrary, no significant modifications were observed in the CG for any of the outcomes. Accordingly, between-group analyses demonstrated that all outcomes showing improvement in the TG were significantly different at post-intervention compared to the CG. Similarly, after the second phase, the CG showed comparable results, except for a non-significant change in MVIC, while muscle histological characteristics were not assessed.

The subsequent analysis conducted by Horwath et al. (2025) explored the muscular degenerative events and muscle regenerative capacity associated with the first phase of the clinical trial of Bankolé et al. through histological evaluation. As described in the previous study, participants were subdivided into TG and CG, and muscle biopsies were taken from the vastus lateralis muscle before and 2 to 7 days after the last completed exercise session. Only within-group comparisons (pre- vs. post-intervention) were analysed.

Histopathological analysis indicated that skeletal muscle was relatively preserved at baseline, with only minor signs of degeneration. Importantly, the training intervention did not exacerbate muscle pathology, as no increases were observed in fibres with centralised nuclei or in the expression of developmental myosin heavy chain. Similarly, markers of immune cell infiltration remained unchanged, suggesting that the intervention did not induce inflammatory responses. In contrast, the training program elicited favourable muscular adaptations, including a statistically significant shift toward larger muscle fibre CSA between 5000 and 6000 µm^2^, consistent with hypertrophy. Moreover, a significant expansion of the satellite cell pool was observed, particularly in fast-twitch fibres, indicating an enhanced regenerative potential. No changes were detected in myonuclear content or telomere length.

#### Aerobic exercise training combined with nutritional intervention

The study by Andersen et al. (2015) investigated the effects of aerobic exercise training combined with post-exercise protein–carbohydrate supplementation in patients with FSHD type 1. In this RCT, 35 patients were allocated to three groups: (1) an AET group receiving a protein–carbohydrate supplement (AET + SUP, *n* = 13; mean age: 42.6 years, 5 females FSHD Clinical Score: 5.2), (2) an AET group receiving a placebo supplement (AET + PLA, *n* = 13; mean age: 45.7 years, 6 females, FSHD Clinical Score: 6.2), and (3) a non-intervention CG (*n* = 9; mean age: 51.3 years, 4 fenales, FSHD Clinical Score: 5.9). The study did not provide information on whether the non-intervention CG maintained their usual physical activity levels or engaged in any physical therapy.

The primary outcomes were fitness, measured as V̇O_2_max, and Wmax during a CPET on a cycle-ergometer, in addition to walking speed obtained from the 6MWT, and a series of secondary outcomes as described below: 5STS, 14-step-stair-test performed first at usual pace and then as fast as possible, standing balance test, self-assessed physical questionnaire, and muscle strength (knee and elbow flexion and extension) measured with a dynamometer testing box. Moreover, SF-36 and daily activity with an accelerometer were monitored. The safety of the protocol was assessed with plasma-CK, as a marker of muscle damage, collected at baseline, at + 3 weeks, at + 7 weeks and at + 12 weeks; however, specific daily time and precautions before collecting the sample were not available.

The intervention consisted of AET performed on an upright stationary bicycle three times per week for 12 weeks. Exercise duration was progressively increased from 15 min during the first week to 20 min during the second week, reaching 30 min per session thereafter, with an intensity corresponding to approximately 70% of V̇O₂max. In addition to exercise, participants in the supplementation group consumed a protein–carbohydrate drink immediately after each training session, providing 163 kcal (23 g whey protein and 17 g carbohydrates), whereas the placebo group received a non-caloric supplement.

Results show that the training groups, merged (AET + SUP and AET + PLA), had a statistically significant augmentation in V̇O_2_max, Wmax and walking speed, while CG had not. Additionally, functional tests, such as the stand-balance test, stair test at self- and fast pace, chair test at fast pace, along with muscle strength tests, were not significantly changed in either the AET(SUP + PLA) or the CG. Authors reported that the AET + SUP did not improve V̇O_2_max, Wmax or walking speed further than AET + PLA. Regarding safety, no episodes of muscle damage were identified by CK during the trial.

### Non-randomized studies

#### Aerobic exercise training

The first study ever implementing an AET intervention in patients with FSHD was conducted by Olsen et al. (2005) using a non-equivalent groups design including both patients and HCs. The study aimed to evaluate the safety and effects of a low-intensity AET program in this population. A total of 8 patients with FSHD (mean age 36 years; 4 females) and 7 age- and sex-matched HCs participated in the intervention. The outcomes assessed at pre- and post-intervention included V̇O₂max and Wmax assessed during a CPET on a cycle ergometer, along with muscle histological characteristics, including fibre type distribution, muscle fibre CSA, and capillary density. Furthermore, exclusively in patients, serum CK levels were collected. The latter, aiming to explore training-induced muscle damage, was obtained at baseline, 3, and 6 weeks, but not at the end of the intervention. The timing of plasma CK sampling relative to exercise was not reported.

The intervention consisted of 12 weeks of cycle-ergometer aerobic exercise training (AET), with frequency progressively increased over the first 4 weeks to five sessions per week (44 sessions in total). Sessions lasted 35 min and were performed at 65% of V̇O₂max.

Following the intervention, significant improvements were observed in the FSHD group in both V̇O₂max and Wmax, with similar ameliorations also reported in HCs. In contrast, no significant changes were detected in muscle histological parameters (fibre type distribution, muscle fibre size, and capillary density) in either group. From a safety perspective, CK levels remained stable throughout the intervention and showed a tendency to decrease over time.

#### Combined aerobic and anaerobic exercise training

The study by Prieur-Blanc et al. (2024) investigated the effects on fitness and walking capacity of a rehabilitation program combining AET and strength domains, both combined with standard occupational therapy, in a cohort of 10 patients with genetically confirmed FSHD (*n* = 10; mean age 50.7 ± 15.2 y; 7 females).

Main outcomes were aerobic performance, evaluated by V̇O_2_max and MAP during a CPET on a cycler-ergometer; walking performance, assessed using the 6MWT and the 10-m walk test (10mWT); and muscle strength, evaluated using an isokinetic test. Moreover, the Insomnia Severity Index (ISI) was used to investigate the quality of sleep, and a visual number scale was used to measure physical fatigue. Assessments were performed at pre- and post-intervention.

The intervention was implemented either as an inpatient program comprising five weekly sessions over four weeks or as a day-care regimen with two to three sessions per week over eight weeks, resulting in the same total number of sessions. The AET component involved 30 min of cycling on a stationary ergometer at an intensity set at the first ventilatory threshold. RT was performed using an isokinetic dynamometer and consisted of four sets totalling 32 repetitions across multiple angular velocities (180°/s, 120°/s, and 60°/s). The initial workload was set at 40% of maximal concentric strength, with 45 s of rest between sets, and was progressively increased by 10% every five sessions until reaching 70% by the end of the program. In addition, conventional therapy included one hour of supervised upper-limb strengthening and joint mobilisation led by an occupational therapist, followed by an additional hour of similar exercises conducted in a balneotherapy environment.

Following the intervention, V̇O₂max and MAP significantly increased. Functional performance also improved, with a significant amelioration in the 6MWT and 10-mWT. In terms of muscle strength, a significant time effect was observed only for knee flexors at 60°/s, whereas no significant changes were detected for knee flexors at 120°/s and 180°/s, along with knee extensors at each angular velocity. Patient-reported outcomes showed a marked reduction in physical fatigue, as measured by the visual number scale, as well as a significant improvement in sleep quality, reflected by a decrease in the ISI score.

### Case studies

#### Combined aerobic and anaerobic exercise training

In the study by Calisgan et al. (2019), a 26-year-old patient with FSHD underwent a multimodal rehabilitation program combining stretching, strength (targeting cervical and respiratory muscles), endurance, and aerobic exercises, performed as 3 sets of 10 repetitions, three times per week for 4 months. The intervention was complemented by weekly kinesiotaping aimed at improving foot drop, balance, and walking capacity. However, the exercise protocol was not described in sufficient detail, as no information was provided regarding the time allocated to each component or the specific exercise modalities employed. This limits the possibility of contextualizing the intervention within the broader literature and, therefore, it will not be included in the methodological comparison with the other studies in the Discussion section.

Outcomes included upper extremity range of motion (ROM), as well as muscle strength of both upper and lower limbs, assessed using the Medical Research Council Scale. Walking capacity was evaluated through the 10mWT and by measuring stair ascent and descent times, both with and without assistance. Fatigue levels were assessed using the FSS. The results indicated overall improvements in muscle strength in both the upper and lower extremities, as well as increased range of motion in the upper limbs. Walking capacity and gait speed also improved, together with functional task performance. Finally, FSS scores improved.

#### Combined aerobic and anaerobic exercise training combined with nutritional intervention

Two case reports reported the effectiveness of a combined aerobic–anaerobic intervention with concomitant nutritional interventions (Pasotti et al. 2014; Quintiero et al. 2025a).

The case study by Pasotti et al. (2014) explored the effects of a controlled exercise training and nutritional supplements in a 43-year-old female wheelchair patient with genetically confirmed FSHD (FSHD score: 12).

Main outcomes (pre- vs. post-intervention) included resting energy expenditure (REE), assessed by indirect calorimetry; endurance performance, evaluated as V̇O₂ at 7.5 W and ventilation exchange (VE) during an incremental stepwise ramp test on a handbike; isometric strength (MVIC), measured using a dynamometer; and pulmonary function tests (PFTs), assessed through spirometry. In addition, routine blood parameters and urine analyses were collected, along with body composition collected through a bioimpedance analysis (fat mass (FM) and fat-free mass (FFM)).

The program integrated AET and strength training with respiratory exercises and nutritional supplementation. The aerobic component consisted of arm cycling performed twice weekly over a 24-week period, with session duration progressively increased from 10 to 20 min by week 13, at an intensity corresponding to 4 kcal per minute (130 bpm). Additionally, bi-weekly strength training was conducted using elastic bands, each including two sets of eight repetitions at an intensity of 50–60% of maximal voluntary contraction, separated by 5 min of rest. Respiratory muscle training involved isocapnic hyperpnea performed four times per week in 2-minute bouts at 50% of vital capacity and a frequency of 20 breaths per minute, using a dedicated device. The nutritional intervention included daily supplementation with branched-chain amino acids (0.1 g·kg⁻¹·day⁻¹, with a 2:1:1 leucine: isoleucine: valine ratio), creatine monohydrate (0.1 g/day), and conjugated linoleic acid (2.4 g/day).

Following the intervention, reductions in V̇O₂ and minute ventilation (V̇E) were observed at 7.5 W during the incremental stepwise ramp test. Muscle strength showed heterogeneous responses, with increases in shoulder abductor strength bilaterally, in right elbow flexors, and in left elbow extensors, whereas a reduction in right elbow flexors, and left elbow extensor. REE remained relatively stable. PFTs remained substantially unchanged, while there was a general amelioration of blood and urine parameters. This was accompanied by a reduction in body mass and FM, and increased FFM.

In the study of Quintiero et al. (2025a), a 19-year-old male FSHD patient, categorized as A category of the Comprehensive Clinical Evaluation Form (CCEF, (Ricci et al. 2016) underwent a personalised nutritional program and exercise for 52 weeks, participating in three evaluation points (baseline, at 6 months, and post-intervention). The main aim was to evaluate the effects of this combined nutritional and PE intervention on body composition, metabolic parameters, perceived physical efficiency, and QoL. Specifically, body composition was assessed with bioelectrical impedance analysis (FFM, body cellular mass (BCM), and FM). Biochemical parameters were collected with blood analyses (blood glucose level, blood insulin level), while metabolic outcomes were measured with indirect calorimetry (resting metabolic rate (RMR). Finally, CIS20R and Functional Assessment Chronic Illness Therapy Fatigue (FACIT-F) were used to evaluate perceived physical efficiency and QoL.

The patient underwent a combined aerobic-strength protocol for a 52-week period, comprising one weekly session of cycling (40–50% of theoretical maximal HR) and two weekly sessions of whole-body strength training targeting core and abdominal muscles, lower-limbs, and upper-limbs (2–4 sets of 10–15 repetitions with 90-second recovery). The macronutrient total daily caloric intake of the nutritional intervention was as follows: 51.2% carbohydrates, 27.7% fats, and 21.1% of proteins (i.e. 1.5 g of proteins/kg of body weight/day) (Quintiero et al. 2025a). Specifically, the protein needs were set according to the nutritional recommendations for the management of sarcopenia (Rogeri et al. 2021). Additionally, muscle protein synthesis was further supported with a nutritional supplementation of 11 g/day of essential amino acids, along with an adequate redistribution of Leucine across the daily meals (1–3 g each meal) (Cereda et al. 2022; Jäger et al. 2017).

Pre vs. post intervention analyses showed consistent improvements across metabolic, body composition, and patient-reported outcomes. Specifically, there was an amelioration in body composition, through an increase in body weight, FFM and BCM, alongside a reduction in FM. Regarding metabolic parameters, blood glucose and insulin levels decreased. In contrast, RMR increased. Finally, CIS20R decreased over time, while FACIT-F improved.

## Discussion

The present review aims to provide an overview of the safety, methodological approaches, effectiveness, and potential risks of exercise interventions in patients with FSHD. Overall, 11 studies were analysed, including a total of 141 patients (86 males, 55 females), with an age range spanning from 20-to-79 years. Among these, 121 patients were classified as FSHD type 1 (Salort-Campana et al. 2020), 1 patient was categorised according to the CCEF as belonging to category A (Quintiero et al. 2025a) and for 19 patients the subtype was not specified (Calisgan et al. 2019; Olsen et al. 2005; Prieur-Blanc et al. 2024). Clinical disease severity was assessed in 57 patients using the Ricci score (Janssen et al. 2016; Voet et al. 2014) and in 48 patients (Andersen et al. 2015, 2017; Pasotti et al. 2014) using the FSHD Clinical Score (Lamperti et al. 2010), with reported values ranging from 0.5 to 4 and from 1 to 12, respectively. Clinical disease severity was not reported in 36 patients (Bankolé et al. 2016; Calisgan et al. 2019; Olsen et al. 2005; Prieur-Blanc et al. 2024; Quintiero et al. 2025a). The main limitation of most studies is the small sample size, which reduces statistical power and limits the robustness and generalizability of the available evidence. This is particularly evident in studies investigating exercise training combined with nutritional intervention. Table 1 reports sample characteristics, intervention types, outcome directions, and key findings of the included studies.

**Table 1:** Description of the studies adopting an aerobic, anaerobic, and combined aerobic-anaerobic exercise training in FSHD patients

Author(s) and years	Subjects	Exercise/nutritional intervention and duration	Main directions of the outcomes	Key findings
**Aerobic exercise training**				
*Randomised controlled trials*				
Voet et al. (2014)	57 FSHD* Type 1 patients subdivided into: a) AET (*n* = 20; 8 F; median age 59 y (21–68); Ricci Score 3.0 (0.5-4.0); duration of illness median 13.0 y (1.0–42.0); ClS-fatigue 41 (35–54)) b) CBT (*n* = 13; 5 F; median age 49 y (24–69); Ricci Score 2.0 (1.0–4.0); duration of illness median 7.0 y (0.0–40.0); ClS-fatigue 43 (35–56)) c) UC (*n* = 24; 7 F; median age 52 y (20–79); Ricci Score 3.0 (1.5-4.0); duration of illness median 16.7 y (1.0–49.0); ClS-fatigue 42 (35–51))	Aerobic training: cycle-ergometer, 3 sessions per week for 16 weeks for a duration of 30 min, with intensity gradually increased from 50% to 65% of HRR. Cognitive-behavioural therapy, a 50-minute lessons directed at insufficient coping with the disease. Usual care No treatment or occasional (conventional) physical therapy.	Between-group changes *Pre- vs. post-intervention (16th weeks)* *Aerobic fitness*: AET = UC CBT = UC *Functional tasks*: AET = UC CBT = UC *Physical activity level*: AET > UC CBT > UC *Fatigue*: AET > UC CBT > UC *Muscle strength*: AET > UC CBT = UC *Pre- vs. post-follow up (28th week)* *Aerobic fitness*: AET = UC CBT = UC *Functional tasks*: AET = UC CBT = UC *Physical activity level*: AET > UC CBT > UC *Fatigue*: AET > UC CBT > UC *Muscle strength*: AET = UC CBT = UC	AET improves muscle strength, physical activity level, and reduces fatigue, but has not effect on aerobic capacity and functional tasks
Janssen et al. (2016)	31 FSHD* Type 1 patients subdivided into: a) AET (*n* = 9; 4 F; mean age 56 ± 15 y; Ricci score: 3.1 ± 1.1) b) CBT (*n* = 9; 3 F; mean age 53 ± 12 y; Ricci Score: 2.3 ± 1.0) c) UC (*n* = 13; 3 F; mean age 53 ± 15 y; Ricci score: 3.0 ± 0.9)	Aerobic training: cycle-ergometer, 3 sessions per week for 16 weeks for a duration of 30 min, with intensity gradually increased from 50% to 65% of HRR. Cognitive-behavioural therapy, a 50-minute lessons directed at insufficient coping with the disease, plus a maximum of 2 60-minute sessions of walking or cycling per day. Usual care No treatment or occasional (conventional) physical therapy.	Between-group changes *Body composition* *(progression of fatty infiltration)*: AET < UC CBT < UC	AET reduces the progression of fatty infiltration in the muscles compared to no exercise
*Randomised controlled trials plus nutritional intervention*				
Andersen et al. (2015)	35 FSHD* 1 Patients subdivided into: a) AET + SUP (*n* = 13; 5 F; mean 42.6 y (24–55); FSHD Clinical Score: 5.2 (3–7)), b) AET + PLA (*n* = 13; 6 F; mean 45.7 y (22–63); FSHD Clinical Score: 6.2 (1–11)) c) CG (*n* = 9; 4 F; mean 51.3 y (24–65); FSHD Clinical Score: 5.9 (2–9))	Aerobic training, cycle-ergometer, 3 sessions per week for 12 weeks, with sessions building up from 15 min (1st week) to 30 min (3rd week and thereafter), with an intensity of 70% V̇O_2_max. Nutritional intervention Protein-Carbohydrate supplement: 163 kcal (23 g whey protein, 17 g carbohydrate, 0 g fat). Placebo: (0 kcal) Control group No nutritional and physical intervention	Between-group changes *Pre- vs. post-intervention (12th week)* AET + SUP did not improve fitness, workload, or walking speed further than AET + PLA. Within-groups changes *Pre- vs. post-intervention (12th week)* *Aerobic fitness and performance*: ↑ AET+(SUP & PLA) = CG *Functional tasks*: ↑ AET+(SUP & PLA) = CG *Physical activity level*: = AET+(SUP & PLA) = CG *Serum creatine kinase*: = AET+(SUP & PLA) *Fatigue*: = AET+(SUP & PLA) *Muscle strength*: = AET+(SUP & PLA) = CG	AET increases safely aerobic fitness and performance, and functional tasks; however, AET plus a protein-carbohydrate supplement does not enhance training adaptations compared to a placebo
*Non-randomised controlled trials*				
Olsen et al. (2005)	8 FSHD* AET (*n* = 8; 4 F; mean age: 36 y (18–55))	Aerobic training, cycle-ergometer, the number of weekly sessions increased during the first 4 weeks until reaching five times/week, totalising 44 sessions in 12 weeks, for a duration of 35 min, with an intensity of 65% V̇O_2_max.	Within-group changes *Pre- vs. post-intervention (12th week)*: ↑*Aerobic fitness and performance*: *= Muscle CSA*: *= Serum creatine kinase*:	AET improves aerobic fitness and performance without muscle damage
**Anaerobic exercise training**				
*Randomized controlled trials*				
Andersen et al. (2017)	12 FSHD* 1 Patients subdivided into: a) Supervised HIT (*n* = 6; 2 F; mean 53 ± 15 y (26–67); Clinical Score: 6.3 ± 4.3 (1–11)) b) UC (*n* = 6; 1 F; mean 46 ± 9 y (32–59); Clinical Score: 7.5 ± 2.1 (5–11))	High-intensity interval training: cycle-ergometer, 3 sessions per week for 8 weeks (supervised) + 8 weeks (unsupervised) for a duration of 21 min, in which two sets of five consecutive 1-min HIT bouts (30 s at low intensity, 20 s at middle-high intensity, and 10 s of all-out, maximal intensity) were performed. Usual care group Not specified	Within-group changes *Pre vs. post-intervention (8th week)* *Aerobic fitness and performance*: ↑ FSHD Supervised HIT = FSHD UC *Functional tasks*: = FSHD Supervised HIT ↑ FSHD UC *Physical activity level*: = FSHD Supervised HIT = FSHD UC *Serum creatine kinase*: = FSHD Supervised HIT *Muscle strength*: = FSHD Supervised HIT = FSHD UC	HIT is feasible and improves aerobic fitness and performance safely
**Combined aerobic-anaerobic exercise training**				
*Randomised controlled trials*				
Bankolé et al. (2016)	16 FSHD* 1 type patients subdivided into: a) TG (*n* = 8; 3 F; mean age 40 ± 13 y) b) CG (*n* = 8; 1 F; mean age 41 ± 9 y) Afterwards, CG was inserted in the intervention as: c) TCG	Combined aerobic/strength exercise, cycle-ergometer, 3 sessions per week for 24 weeks, for a duration of 35 min, of which two sessions consisted of 25-min constant moderate intensity (60% of MAP), followed by 5 sets of 10 near-maximal revolutions, while the third session was composed by five 1-min intervals at 80% MAP.	Within-group changes *Pre- vs. post-intervention (24th week)*: *Aerobic fitness and performance*: ↑ TG = CG ↑ TCG *Functional tasks*: ↑ TG = CG ↑ TCG *Muscle CSA*: ↑ TG = CG *Serum creatine kinase*: = FSHD TG = TCG *Fatigue*: ↓ TG = CG ↓ TCG *Muscle strength*: ↑ TG = CG = TCG	Combined exercise training is safe and effective across multiple domains (aerobic fitness and performance, muscle strength, functional tasks, fatigue, and muscle CSA)
Horwath et al. (2025)	16 FSHD* Type 1 patients subdivided into: a) TG (*n* = 8; 3 F; 40 ± 13 y) b) CG (*n* = 8; 1 F; 44 ± 10 y)	Combined aerobic/strength exercise, cycle-ergometer, 3 sessions per week for 24 weeks, for a duration of 35 min, of which two sessions consisted of 25-min constant moderate intensity (60% of MAP), followed by 5 sets of 10 near-maximal revolutions, while the third session was composed by five 1-min intervals at 80% MAP.	Within-group changes *Muscle CSA*: ↑ TG = CG *Satellite cell pool expansion in fast muscle fibres*: ↑ TG = CG	Combined exercise training promotes expansion of the satellite cell pool within muscle fibres, while markers of immune cell infiltration remain unchanged.
*Non-randomised controlled trials*				
Prieur-Blanc et al. (2024)	10 FSHD* patients (*n* = 10; 7 F, mean age: 50.7 ± 15.2 y)	Rehabilitation program, composed of aerobic, strength, and standard occupational therapy of 5 hospital-based rehabilitation sessions for 4 week. *Aerobic component*: cycle-ergometer for 30 min at an intensity corresponding to the first ventilatory threshold. *Strength training*: isokinetic device for a total of 10 sets at different angular velocities at 40% of the maximum concentric strength and increasing by 10% every five sessions, with a rest period of 45 s. *Conventional therapy*: 1 h of strength training and articular mobilisation of the upper limbs	Within-group changes *Pre- vs. post-intervention (4th week)*: ↑ *Aerobic fitness and performance* ↑ *Functional tasks* ↓ *Fatigue* ↑*Muscle strength*:	Combined exercise training is effective across multiple domains (aerobic fitness and performance, muscle strength, functional tasks, and fatigue)
*Case study*				
Calisgan et al. (2019)	1 FSHD* (*n* = 1; F; 26 y)	Combined exercise training, composed of stretching, strength, endurance, and aerobic exercises, for 3 days per week for 4 months, plus kinesiotape once per week.	Within-patient changes *Pre- vs. post-intervention (24th week)*: ↑ *Functional tasks* ↓ *Fatigue* ↑ *Muscle strength*	Combined exercise training is effective across multiple domains (muscle strength, functional tasks, and fatigue)
*Case study plus nutritional intervention*				
Pasotti et al. (2014)	1 FSHD* (*n* = 1; F; 43 y; FSHD score 12)	Combined endurance and strength training, plus nutritional intervention *Aerobic training*, arm cycle-ergometer for 2 sessions/week for 24 weeks, starting from 10 min and progressively building up to 20 min from week 13 at an intensity of 130 beats per minute. *Strength training*, with an elastic band for 2 sessions/week for 24 weeks, for 2 sets of 8 repetitions with 5 min rest between series at 50–60% of MVC. *Respiratory muscle training*: 4 times/week for 2 min (50% vital capacity at 20 min^− 1^) of isocapnic hyperpnea. *Nutritional intervention* Supplementation was provided with BCAA, creatine monohydrate and CLA	Within-patient changes *Pre- vs. post-intervention (24th week)*: ↑ *Aerobic fitness* ↑ *Body composition* ↑ *Muscle strength*:	Combined exercise training is effective across multiple domains (aerobic fitness, strength, and body composition)
Quintiero et al. (2025)	1 FSHD* (*n* = 1, M, 19 y; subcategory A3)	Combined aerobic-anaerobic exercise *Aerobic training*, cycle-ergometry, one session/week for 52 weeks for 45 min, with an intensity between 40% and 50% of the theoretical HRmax. *Resistance training*, two sessions/week for 52 weeks, for 60 min, performed as 2–4 sets of 10–15 repetitions, with between-sets recovery of 1 min and 30s. *Supplementation* 11 g/day of essential amino acid (Aminotrofic-NE).	Within-patient changes *Pre- vs. post-intervention (52nd week)*: ↑ *Body composition* ↓ *Fatigue*	Combined exercise training is effective across fatigue and body composition

Detailed study characteristics and outcome data are provided in the Supplementary Table 1

*AET* aerobic exercise training; *AET + SUP* aerobic training groups plus supplementation ; *AET + PLA* aerobic training groups plus placebo; *AET+(SUP & PLA)* aerobic training groups plus supplementation and placebo; *BCAA* branched-chain amino acids; *CBT* cognitive-behavioural therapy; *CG* control group; *ClS-fatigue* Checklist Individual Strength – Fatigue; *CLA* conjugated linoleic acid; *CSA* cross-sectional area; *F* females; *FSHD* facioscapulohumeral muscle dystrophy; *n* number; *g* grams; *HC* healthy control; *HIT* high-intensity training; *HRmax* heart rate maximum; *HRR* heart rate reserve; *kcal* kilocalorie; *M* male; *MAP* maximal aerobic power; min minutes; *MVC* maximal voluntary contraction; *Pre* pre-intervention; *post* post-intervention; *s* seconds; *TCG* training control group; *TG* training group; *UC* usual care; *V̇O*_*2*_*max* maximal oxygen uptake; vs. versus; *y* years

↓ indicates a within-group significant reduction. N.B. for case study, indicate a reduction from baseline; **↑** indicates a within-group significant improvement. N.B. for case study, indicate an increase from baseline; = indicates a within-group non-significant modification or a between-group non-significant comparison; > indicates a between-group significant improvement; < indicates a between-group significant reduction; * genetically assessed. Results are reported as mean ± SD unless otherwise specified

### Considerations on aerobic exercise training in FSHD

Among the considered studies, 62 patients underwent AET (Andersen et al. 2015; Janssen et al. 2016; Olsen et al. 2005; Voet et al. 2014), including 13 who also received nutritional supplementation (Andersen et al. 2015).

Across these studies, AET was associated with improvements across a heterogeneous range of outcomes. Evidence from RCTs indicates reduced fatigue accompanied by increased muscle strength and daily activity levels (Voet et al. 2014), a slower progression of fatty infiltration (Janssen et al. 2016), and improvements in aerobic fitness and performance (Andersen et al. 2015). Notably, improvements in aerobic fitness, an adaptation typically expected following AET (Mann et al. 2013), are not consistently observed across RCTs, as Voet et al. (2014) did not report significant changes in this domain. However, this adaptation is supported by findings from a non-randomized study (Olsen et al. 2005).

**Fig. 1:**
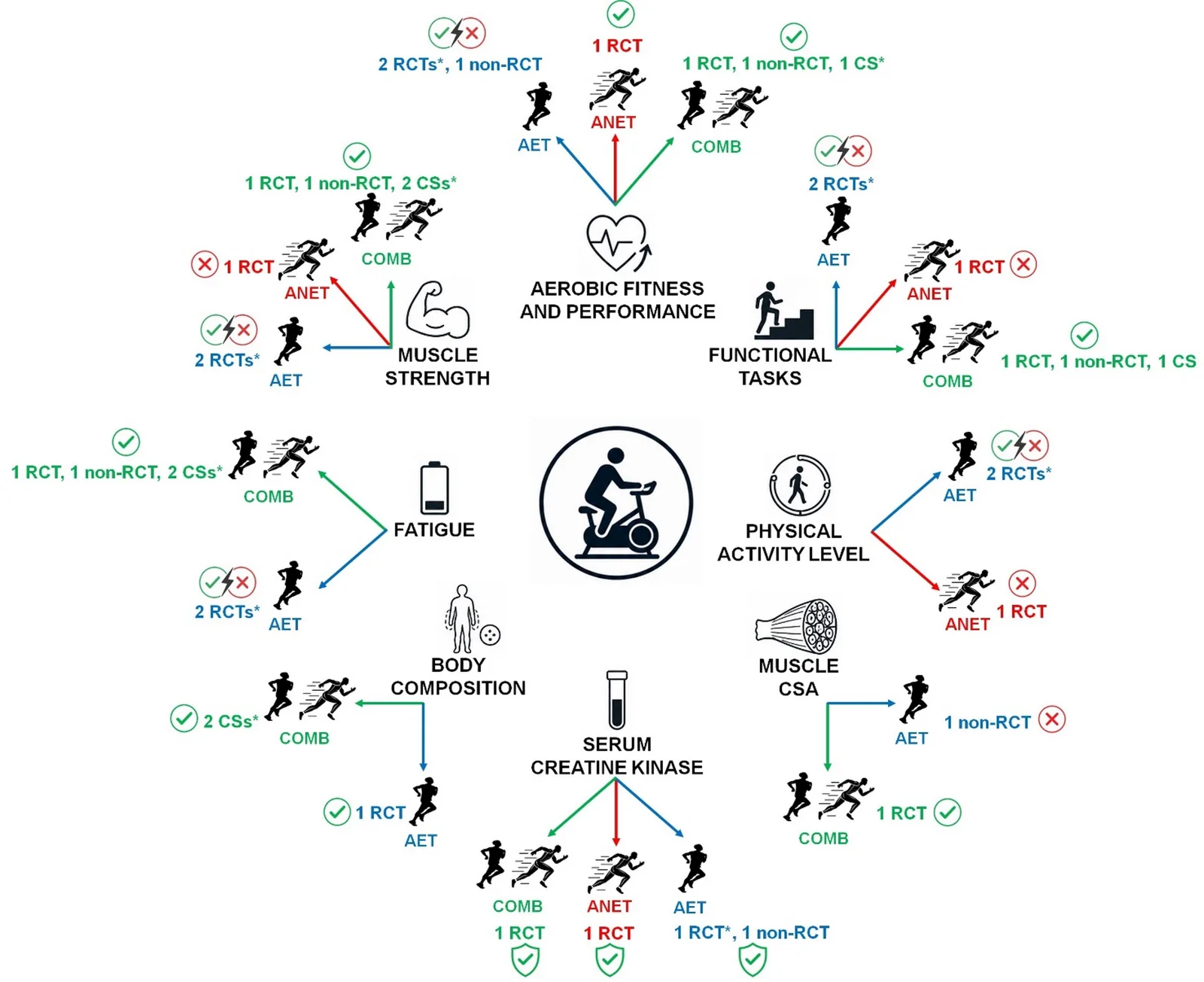
Overview of the effects of different exercise modalities on clinical outcomes in patients with FSHD. Aerobic exercise training (AET), anaerobic exercise training (ANET), and combined exercise training (COMB) are mapped against key outcome domains, including aerobic fitness and performance, muscle strength, functional tasks, physical activity level, muscle cross-sectional area (CSA), serum creatine kinase, body composition, and fatigue. Only outcomes assessed in at least two training modalities are included. Blue, red, and green arrows denote associations with AET, ANET, and COMB, respectively. The number and design of studies contributing to each outcome (randomized controlled trials, RCTs; non-randomized controlled trials, non-RCTs; case studies, CS) are reported alongside the corresponding modality, providing an indication of the level of evidence. Of note, the figure summarizes the direction of effects but does not reflect its magnitude. Importantly, the figure is intended to provide a visual summary of the reported effects and should not be interpreted as a direct comparison between exercise modalities

Overall, despite heterogeneity among studies (Fig. 1), these findings appear clinically relevant, as fatigue and aerobic fitness are key limiting factors in patients with FSHD (Crisafulli et al. 2024a, 2025a; Schipper et al. 2017), while fatty infiltration is a major determinant of functional impairment (Mellion et al. 2022; Skalsky et al. 2008). Collectively, the analysed evidence suggests that AET is a safe and beneficial treatment in this population under the investigated conditions, although the small sample sizes limit the strength and generalizability of these inferences.

All studies used cycle ergometry, likely reflecting balance impairments in patients with FSHD (Crisafulli et al. 2025b; Rijken et al. 2014) and the reduced postural demands compared with weight-bearing modalities such as treadmill walking (Pang et al. 2006), although this rationale is not consistently reported. Training frequency ranged from three weekly sessions (Andersen et al. 2015; Voet et al. 2014) to up to five in Olsen et al. (2005), although initial training load was not specified. Session duration was relatively consistent across studies, with protocols targeting approximately 30 min per session.

Instead, prescription of exercise intensity was less uniform, with two studies (Janssen et al. 2016; Voet et al. 2014) based on HRR % (same cohort), and two studies (Andersen et al. 2015; Olsen et al. 2005) utilizing V̇O2max %. Of note, both approaches are well-established and widely used in the exercise prescription literature. However, such relative-intensity methods are based on maximal HR and V̇O₂ values, which may not be achievable in some patients due to muscle weakness and exercise intolerance (Vera et al. 2022). Moreover, they may poorly account for interindividual variability in metabolic responses to exercise (Iannetta et al. 2020; Mann et al. 2013), as well as for altered fatigue dynamics in neuromuscular disorders (Voet et al. 2022), potentially resulting in misclassification of exercise intensity and contributing to suboptimal or heterogeneous training stimuli across participants. Importantly, this methodological aspect may partly explain the lack of improvement in aerobic fitness reported by Voet et al. (2014). In contrast, AET prescribed based on ventilatory thresholds has been shown to attenuate interindividual variability compared with relative percentage-based methods (Mann et al. 2013) and to induce significantly greater improvements in aerobic fitness (Wolpern et al. 2015). In the context of FSHD, this approach has been implemented only in the study by Prieur-Blanc et al. (2024). However, it was applied within a combined, non-RCT training intervention, thereby limiting the ability to isolate the specific contribution of such approach.

Based on the screened literature, AET in patients with FSHD appears to be a useful intervention with the potential to exert beneficial effects across multiple domains. Given that ventilatory threshold-based AET prescription has been shown to provide a more precise and effective approach to exercise intensity regulation than traditional percentage-based methods, its application in patients with FSHD warrants investigation in well-controlled studies.

### Considerations on anaerobic exercise training in FSHD

Data on ANET in FSHD resulted the most limited. Only one RCT study met the inclusion criteria (Andersen et al. 2017), comprising a total of 12 patients. The reasons for this discrepancy compared with the larger body of evidence on AET remain unclear. However, it is plausible that the precautionary approach that has historically guided clinical practice, driven by concerns regarding overwork weakness (Johnson and Braddom 1971), may have also shaped the research landscape, limiting investigation of high-intensity approach as perceived like potentially harmful.

Whatever the case, the results of the available study suggest that this training modality may improve aerobic fitness and performance. Besides, no clear effect on muscle strength, which would be expected following HIT (Alzar-Teruel et al. 2022; Caparrós-Manosalva et al. 2023), or on functional outcomes were described. This finding appears consistent with a Cochrane review by Voet et al. (2019), reporting that strength training alone may have little or no effect in patients with muscle diseases. The mechanisms underlying the absence of specific strength gains in FSHD remain unclear. However, it may be speculated that the preferential involvement of type II muscle fibres, as reported in both murine FSHD models and human studies (D’Antona et al. 2007; Lassche et al. 2013) may contribute to this pattern, given their key role in anaerobic performance (Esbjörnsson et al. 1993), potentially attenuating the expected training responses.

As an important methodological consideration, although HIT can improve strength, it may be less effective than traditional resistance training (Hung et al. 2025). Therefore, it may also be speculated that the training stimulus was insufficient to produce measurable gains in muscle strength.

In summary, evidence on ANET in FSHD remains insufficient to draw any conclusion. The available data may suggest potential benefits for improvements in performance and aerobic fitness, but not for muscle strength or functional outcomes. Future studies in larger cohorts will be required to confirm or refute these findings and potentially identify factors useful for optimization of training protocols.

### Considerations on combined exercise training in FSHD

Based on the body of literature considered, data on combined training interventions include a total of 29 patients (Bankolé et al. 2016; Calisgan et al. 2019; Pasotti et al. 2014; Prieur-Blanc et al. 2024; Quintiero et al. 2025a). Compared with the studies discussed above, such training protocols are characterized by greater heterogeneity, both in the outcomes investigated and in their methodological design.

From a methodological standpoint, the aerobic component implemented across the included studies shows both shared and divergent features compared with protocols specifically based on AET. Cycle ergometry further appears to be the predominant modality, except for the case report by Pasotti et al. (2014), in which an arm ergometer was employed due to the patient’s inability to use the lower limbs. The method used to prescribe exercise intensity represents the most heterogeneous aspect. Bankolé et al. (2016) based intensity on a percentage of MAP, Prieur-Blanc et al. (2024) used the first ventilatory threshold, Quintiero et al. (2025a) prescribed intensity as a percentage of HR max, whereas Pasotti et al. (2014) does not specify the rationale.

About the anaerobic component, Bankolé et al. (2016) was the only to implement a HIT protocol, similarly to the ANET protocol by Andersen et al. (2017). By contrast, the remaining studies employed resistance training interventions. Also in this case, a wide heterogeneity is observed in exercise intensity prescription. Two studies (Pasotti et al. 2014; Prieur-Blanc et al. 2024) based their approach on maximal voluntary contraction (MVC). Bankolé et al. (2016), in line with the rationale adopted for the aerobic component, prescribed intensity relative to MAP, albeit at higher percentages. In contrast, Quintiero et al. (2025a) does not clearly specify a defined criterion for determining the ANET intensity.

Nevertheless, all the analyzed studies consistently report beneficial effects. The only RCT (Bankolé et al. 2016) showed improvements in aerobic fitness and performance outcomes, alongside positive effects on muscle endurance, walking-related measures, muscle strength, and reductions in perceived fatigue. Consistent findings are also reported in non-randomized studies, particularly with respect to aerobic fitness and performance (Pasotti et al. 2014; Prieur-Blanc et al. 2024), walking-related outcomes (Calisgan et al. 2019; Prieur-Blanc et al. 2024), muscle strength (Calisgan et al. 2019; Pasotti et al. 2014; Prieur-Blanc et al. 2024), and perceived fatigue (Calisgan et al. 2019; Prieur-Blanc et al. 2024; Quintiero et al. 2025a). In addition, case reports combining exercise with nutritional interventions have indicated improvements across multiple body composition parameters (Pasotti et al. 2014; Quintiero et al. 2025a).

When examining approaches, combined training appears to be the most effective modality for improving walking-related parameters, as all studies in which these outcomes were assessed, comprising a RCT (Bankolé et al. 2016), a non-randomized study (Prieur-Blanc et al. 2024), and a case study (Calisgan et al. 2019) consistently reported improvements, whereas AET findings are heterogeneous (Andersen et al. 2015; Voet et al. 2014), and no improvements were observed following ANET (Andersen et al. 2017). Another relevant observation concerns muscle strength, for which a similar pattern emerges. Consistent improvements are reported across all studies evaluating this outcome within combined training protocols (Bankolé et al. 2016; Calisgan et al. 2019; Pasotti et al. 2014; Prieur-Blanc et al. 2024). In contrast, AET shows heterogeneous findings, with improvements reported by Voet et al. (2014) but not by Andersen et al. (2015), while no improvements are observed following ANET (Andersen et al. 2017). Of note, some protocols incorporated overlapping training characteristics, particularly when combined interventions included high-intensity interval components. Therefore, the observations discussed above should be interpreted cautiously, particularly in the absence of direct head-to-head studies. Figure 1 presents a graphical representation of the effects associated with specific training interventions.

 = Beneficial effect (consistent findings across studies, when available).

 = Absence of effect (consistent findings across studies, when available).

= Inconsistent findings across studies.

 = Absence of muscle damage.

Asterisk (*): presence of studies including nutritional intervention.

The small sample sizes and the heterogeneity of the included study designs preclude definitive conclusions. However, the consistency of findings across studies (Fig. 1) raises the hypothesis that combined training may have the potential to elicit broad multi-domain adaptations. Whether this potential differs from that of AET or ANET alone remains unknown, as no study to date has directly compared these approaches using shared outcome measures. This may represent a valuable matter for future studies.

### Considerations on exercise training combined with nutritional intervention

Within the scope of the identified literature, the evidence on exercise training combined with nutritional interventions is quite limited, comprising only one RCT (Andersen et al. 2015) and two case reports (Pasotti et al. 2014; Quintiero et al. 2025a), for a total of 15 patients.

By design, case reports cannot provide meaningful inference regarding the added value of nutritional strategies over exercise alone and therefore offer limited insight for this specific question. Nevertheless, the improvements in body composition reported across these studies are of interest, given its central role in maintaining physical function in FSHD (Crisafulli et al. 2024b; Skalsky et al. 2008). Notably, AET alone has also been shown to elicit favourable effects on body composition, particularly through a reduction in the rate of muscle fat infiltration (Janssen et al. 2016). Future studies should further investigate whether combined interventions may be more beneficial on body composition than exercise training alone, as suggested in other populations (Foster-Schubert et al. 2012; Xie et al. 2024).

RCT findings (Andersen et al. 2015) suggest that post-exercise protein–carbohydrate supplementation does not confer additional benefits to AET compared with placebo. However, this study did not consider habitual dietary protein intake, nor the adequacy of energy intake relative to individual requirements, introducing two potential sources of bias.

First, evidence from healthy and physically active populations indicates that the effectiveness of protein intake on training adaptations is primarily driven by total daily intake rather than nutrient timing per se (Jäger et al. 2017; Lak et al. 2024). For instance, in endurance athletes, protein intake is typically around ~ 1.5 g·kg of body mass (BM)⁻¹·day⁻¹, with intakes up to ~ 2.0 g·kgBM⁻¹·day⁻¹ to support recovery, adaptive responses to training, and performance (Witard et al. 2025). Although these values cannot be directly extrapolated to patients with FSHD, they highlight the relevance of total dietary protein intake when interpreting supplementation strategies, to reduce heterogeneity in overall protein intake. Moreover, both under-feeding (Carbone et al. 2012) and over-feeding (Bouchard et al. 2014) can affect body composition and performance, representing an additional key factor that should be standardized when evaluating the effects of supplementation. These methodological limitations may therefore have, at least in part, reduced the ability to detect potential effects of supplementation beyond exercise-induced adaptations. Accordingly, future studies should standardize both diet daily protein intake and total energy balance to allow a clearer evaluation of supplementation effects.

Besides, literature evidence suggests that the distribution of protein-derived leucine across the day may also influence muscle protein synthesis. Specifically, a per-meal leucine intake of approximately 1–3 g is considered sufficient to maximize muscle protein synthesis in both healthy and sarcopenic populations (Jäger et al. 2017; Cereda et al. 2022), with no additional benefits at higher intakes (Glynn et al. 2010). This is particularly relevant given the association between muscle mass and physical performance in FSHD (Skalsky et al. 2008; Leung et al. 2015).

Noteworthy, preliminary data supporting a nutritional strategy based on the accounting of energy balance, overall protein intake, and leucine distribution is provided by the case report of Quintiero et al. (2025a), where one year of intervention, combined with exercise training, was associated with improvements in metabolic health, body composition, and patient-reported outcomes. In the absence of specific nutritional guidelines for FSHD (Quintiero et al. 2025b), this integrated framework may represent a useful direction for future intervention studies.

### Safety profile of exercise in FSHD

Based on the studies considered in this review, PE seems feasible and beneficial in patients with FSHD and, under the experimental conditions described, no study has reported overwork weakness. However, it should be noted that the included studies predominantly involved ambulatory patients and, when disease severity was reported, generally enrolled individuals with mild-to-moderate impairment. Notable exceptions include the case report by Pasotti et al. (2014), describing a non-ambulatory patient with an FSHD Clinical Score of 12, and the studies by Andersen et al. (2015, 2017), which included patients with FSHD Clinical Scores up to 11 (Table 1), values indicative of a severe form of disease according to the original scale description (Lamperti et al. 2010). Consequently, the favorable adaptations and apparent safety profile are likely to be most representative of patients with preserved ambulation and less advanced disease stages, whereas evidence in severely affected individuals remains comparatively scarce. Therefore, caution is warranted when extrapolating these findings to patients with advanced functional impairment.

As a marker of exercise-induced muscle damage, several studies (Andersen et al. 2017; Andersen et al. 2015; Bankolé et al. 2016; Olsen et al. 2005) have assessed plasma CK levels, a parameter commonly used across several myopathies (Crisafulli et al. 2025c; Mah et al. 2014). No episodes of hyperCKemia were reported. However, with the exception of Bankolé et al. (2016), these studies did not specify the timing of CK assessment relative to exercise sessions, thereby limiting the interpretability of these findings (Brancaccio et al. 2007; Crisafulli et al. 2024c). Furthermore, CK levels in myopathies primarily reflect sarcolemmal disruption (Hord et al. 2025; Ozawa et al. 2001), which appears to be less prominent in FSHD than in Duchenne muscular dystrophy and other dystrophies (Bittel et al. 2020; Nigro and Piluso 2015). Consequently, CK may have limited sensitivity as a marker of exercise-induced muscle damage in this population. In addition, the effect of training on inflammation has been investigated in only one study (Horwath et al. 2025), which reported no increase in muscle inflammatory infiltrates following combined training, further supporting its safety profile.

However, despite encouraging evidence, objective criteria to guide safe exercise prescription in these patients are still lacking. It remains unclear whether baseline muscle status at the onset of training is a relevant determinant of risk. This is indirectly supported by preclinical evidence: Fowler and Taylor (1982), in dystrophic animal models (not FSHD-specific), reported that exercise does not accelerate muscle damage when initiated at earlier or less severe disease stages, whereas it may exacerbate atrophy in more advanced conditions. Although these findings have clear limitations in terms of direct translatability, they suggest that baseline muscle status could influence the risk profile of exercise interventions. At this stage, this remains speculative but may represent a relevant direction for future research with potential implications for exercise safety in FSHD.

Additionally, future studies may benefit from incorporating a broader set of safety-related outcomes, including symptom exacerbation, fatigue, pain, and functional responses, which are commonly used in neuromuscular exercise studies (Voet et al. 2019), alongside imaging and inflammatory markers, which are used to monitor disease involvement and progression in FSHD (Dahlqvist et al. 2020). Such a multidimensional assessment may provide a more robust characterization of exercise-related safety and potential adverse effects in these patients.

## Future directions in exercise-based management of FSHD: toward patient stratification, long-term outcome evaluation, and detection of overwork weakness

Beyond the methodological gaps already discussed, the growing evidence supporting the feasibility and benefits of PE in FSHD is still hindered in its translation into practice by key factors. In particular, to address the marked clinical variability of FSHD, the adoption of consistent and shared criteria for patient stratification in exercise studies appears essential, yet as shown, is currently lacking. Future studies should therefore aim to implement stratified designs, for example by grouping patients based on clinical phenotype (e.g. CCEF categories), disease severity, or muscle involvement patterns, and by reporting subgroup-specific responses to interventions. Importantly, Bettio et al. (2024) reported that exercise performed at a young age is associated with different clinical manifestations across categories defined by the CCEF (Bettio et al. 2024). This finding suggests that exercise effects may vary according to clinical phenotype, warranting targeted investigations to better delineate patient-specific responses and optimize intervention strategies. Similarly, it will be important to clarify whether disease stage, quantified using clinical scales, imaging techniques, or both (Vincenten et al. 2025), at the time of training influences both the magnitude of exercise-induced adaptations and the risk of adverse outcomes.

Beyond stratification, a key challenge lies in the development of personalized exercise prescription strategies. Current approaches largely rely on standardized protocols and relative intensity measures, which insufficiently account for interindividual variability in physiological capacity, fatigue responses, and adaptive reserve. Future studies should therefore move toward individualized prescription models by:

- integrating physiological markers (e.g. ventilatory thresholds, submaximal responses),
- incorporating patient-reported outcomes (e.g. fatigue patterns, perceived exertion),
- and evaluating adaptive responses over time to enable dynamic adjustment of training load.

In clinical practice, patients with FSHD often operate near their maximal functional capacity in daily life, meaning that identical prescribed exercise intensities may elicit markedly different physiological and functional loads. Capturing this variability should therefore be a key focus of future research. Importantly, exercise does not occur in isolation but interacts with daily activities, environmental demands, and behavioral factors. Future studies should integrate real-world data collection, such as wearable activity monitoring, digital diaries, or registry-based data, to quantify the cumulative daily load (including work, mobility, and participation). This would improve ecological validity and may help explain inter-individual variability in training responses while preventing unintended overload.

Uncertainty also remains regarding long-term effects. Future research should prioritize longitudinal designs, including prospective cohort studies and registry-based follow-up, to compare disease progression, functional trajectories, and participation outcomes between physically active and less active patients over time.

Finally, the occurrence of overwork weakness, not reported in the studies reviewed, remains an important issue. While this may reflect the use of safe and controlled exercise protocols in research settings, it also highlights the difficulty of systematically capturing such events in experimental designs. Clinical surveillance is therefore essential. Patient registries (Banerji et al. 2020; Bettio et al. 2021; Guien et al. 2018) may provide a valuable tool to systematically capture potential cases of overwork weakness. Future studies should embed standardized registry-based data collection, including type, intensity, and cumulative physical activity load, recovery patterns, symptom fluctuations, and adverse events. Standardizing these variables will be essential to identify activity patterns associated with increased risk and to improve the safety of exercise prescription in clinical practice.

## Conclusion

The analyzed evidence supports PE as a generally safe intervention in FSHD, with consistent benefits emerging for AET and combined training modalities. Although evidence on anaerobic approaches remains limited, emerging findings suggest potential that warrants further investigation. At the same time, the field is evolving from a cautious, safety-driven perspective toward a more proactive approach in which exercise is increasingly recognized as a meaningful component of care. While the current literature is characterized by relatively small sample sizes and methodological heterogeneity, these challenges also reflect the complexity and variability inherent to FSHD. Future progress will depend on strengthening methodological consistency, embracing patient stratification, and advancing personalized and context-aware exercise prescription. By integrating physiological, functional, and real-world data, the field is well positioned to move toward more individualized, evidence-informed strategies that better reflect the needs and daily realities of patients with FSHD.

## Supplementary Information

Below is the link to the electronic supplementary material.

Supplementary Material 1

## Data Availability

Not applicable.

## Abbreviations

10mWT: 10-m walk test
5STS: 5-time sit-to-stand test
6MWT: 6-minute walking test
AET: Aerobic exercise training
AET + PLA: Aerobic exercise training plus placebo supplement
AET + SUP: Aerobic exercise training plus a protein–carbohydrate supplement
ANET: Anaerobic exercise training
BCM: Body cellular mass
CBT: Cognitive-behavioural training
CCEF: Comprehensive clinical evaluation form
CG: Control group
CIS-fatigue: Checklist individual strength fatigue subscale
CK: Creatine kinase
CPET: Cardiopulmonary exercise test
CSA: Cross-sectional area
FACIT-F: Functional assessment chronic illness therapy fatigue
FFM: Fat-free mass
FM: Fat mass
FSHD: Facioscapulohumeral muscular dystrophy
FSS: Fatigue severity scale
HC: Healthy controls
HIT: High-intensity interval training
HRmax: Maximum heart rate
HRR: Heart rate reserve
ISI: Insomnia severity index
MAP: Maximal aerobic power
MRI: Magnetic resonance imaging
MVIC: Maximal voluntary isometric contraction
PE: Physical exercise
PFT: Pulmonary function tests
QoL: Quality of life
RCT: Randomized controlled trials
REE: Resting energy expenditure
ROM: Range of motion
SF-36: Short form health survey-36
TG: Training group
UC: Usual care
VE: Ventilation exchange
V̇O₂max: Maximal oxygen uptake
V̇O₂peak: Peak oxygen uptake

## References

[CR1] Alzar-Teruel M, Aibar-Almazán A, Hita-Contreras F, Carcelén-Fraile MDC, Martínez-Amat A, Jiménez-García JD, Fábrega-Cuadros R, Castellote-Caballero Y (2022) High-intensity interval training among middle-aged and older adults for body composition and muscle strength: A systematic review. Front Public Health 10:992706. 10.3389/fpubh.2022.992706 36249241 PMC9557068

[CR3] Andersen G, Prahm KP, Dahlqvist JR, Citirak G, Vissing J (2015) Aerobic training and postexercise protein in facioscapulohumeral muscular dystrophy: RCT study. Neurology 85(5):396–403. 10.1212/WNL.0000000000001808 26156512

[CR2] Andersen G, Heje K, Buch AE, Vissing J (2017) High-intensity interval training in facioscapulohumeral muscular dystrophy type 1: a randomized clinical trial. J Neurol 264(6):1099–1106. 10.1007/s00415-017-8497-9 28470591

[CR4] Ansari U, Nadora D, Ong L, Sedighi R, Tabaie E, Ansari Z, Alam M, Syed B, Amani N, Preiss-Farzanegan S (2025) Current landscape for the management of facioscapulohumeral muscular dystrophy and emerging treatment modalities: a literature review. AIMS Neurosci 12(2):291–311. 10.3934/Neuroscience.2025016 PMC12287641 40717734

[CR101] Banerji CRS, Cammish P, Evangelista T, Zammit PS, Straub V, Marini-Bettolo C (2020) Facioscapulohumeral muscular dystrophy 1 patients participating in the UK FSHD registry can be subdivided into 4 patterns of self-reported symptoms. Neuromuscul Disord 30(4):315–328. https://doi.org/10.1016/j.nmd.2020.03.001 10.1016/j.nmd.2020.03.001 32327287

[CR5] Bankolé LC, Millet GY, Temesi J, Bachasson D, Ravelojaona M, Wuyam B, Verges S, Ponsot E, Antoine JC, Kadi F, Féasson L (2016) Safety and efficacy of a 6-month home-based exercise program in patients with facioscapulohumeral muscular dystrophy: A randomized controlled trial. Med (Baltim) 95(31):e4497. 10.1097/md.0000000000004497 PMC4979851 27495097

[CR7] Bettio C, Salsi V, Orsini M, Calanchi E, Magnotta L, Gagliardelli L, Kinoshita J, Bergamaschi S, Tupler R (2021) The Italian National Registry for FSHD: an enhanced data integration and an analytics framework towards Smart Health Care and Precision Medicine for a rare disease. Orphanet J Rare Dis 16(1):470. 10.1186/s13023-021-02100-z 34736505 PMC8567605

[CR6] Bettio C, Banchelli F, Salsi V, Vicini R, Crisafulli O, Ruggiero L, Ricci G, Bucci E, Angelini C, Berardinelli A, Bonanno S, D’Angelo MG, Di Muzio A, Filosto M, Frezza E, Maggi L, Mongini T, Pegoraro E, Rodolico C, Tupler R (2024) Physical activity practiced at a young age is associated with a less severe subsequent clinical presentation in facioscapulohumeral muscular dystrophy. BMC Musculoskelet Disord 25(1):35. 10.1186/s12891-023-07150-x 38183077 PMC10768364

[CR8] Bittel AJ, Sreetama SC, Bittel DC, Horn A, Novak JS, Yokota T, Zhang A, Maruyama R, Rowel QLK, Jaiswal JK, Chen YW (2020) Membrane repair deficit in facioscapulohumeral muscular dystrophy. Int J Mol Sci 21(15). 10.3390/ijms21155575 PMC7432481 32759720

[CR9] Bouchard C, Tchernof A, Tremblay A (2014) Predictors of body composition and body energy changes in response to chronic overfeeding. Int J Obes (Lond) 38(2):236–242. 10.1038/ijo.2013.77 23736367 PMC3773296

[CR10] Brancaccio P, Maffulli N, Limongelli FM (2007) Creatine kinase monitoring in sport medicine. Br Med Bull 81–82:209–230. 10.1093/bmb/ldm014 17569697

[CR11] Buchheit M, Laursen PB (2013) High-intensity interval training, solutions to the programming puzzle: Part I: cardiopulmonary emphasis. Sports Med 43(5):313–338. 10.1007/s40279-013-0029-x 23539308

[CR12] Calisgan E, Yilmaz O, Tuncel D, Duger T (2019) Early adulthood onset of muscle weakness in facioscapulohumeral muscular dystrophy and physical therapy management: An unusual case report. Medicine 8(4):1036–1040

[CR13] Caparrós-Manosalva C, Garrido-Muñoz N, Alvear-Constanzo B, Sanzana-Laurié S, Artigas-Arias M, Alegría-Molina A, Vidal-Seguel N, Espinoza-Araneda J, Huard N, Pagnussat AS, Sapunar J, Salazar LA, Marzuca-Nassr GN (2023) Effects of high-intensity interval training on lean mass, strength, and power of the lower limbs in healthy old and young people. Front Physiol 14:1223069. 10.3389/fphys.2023.1223069 37829114 PMC10565117

[CR14] Carbone JW, McClung JP, Pasiakos SM (2012) Skeletal muscle responses to negative energy balance: effects of dietary protein. Adv Nutr 3(2):119–126. 10.3945/an.111.001792 22516719 PMC3648712

[CR15] Cereda E, Pisati R, Rondanelli M, Caccialanza R (2022) Whey protein, leucine- and vitamin-D-enriched oral nutritional supplementation for the treatment of sarcopenia. Nutrients 14(7). 10.3390/nu14071524 PMC9003251 35406137

[CR16] Cerqueira É, Marinho DA, Neiva HP, Lourenço O (2019) Inflammatory effects of high and moderate intensity exercise-a systematic review. Front Physiol, *10*, 1550. 10.3389/fphys.2019.01550 PMC6962351 31992987

[CR100] Crisafulli O, Baptista R, Drid P, Grattarola L, Bottoni G, Lavaselli E, Negro M, Tupler R, Quintiero V, D’Antona G (2025a) Analysis of body fluid distribution, phase angle and its association with maximal oxygen consumption in facioscapulohumeral dystrophy: an observational study. Health Sci Rep 8(1):e70335. https://doi.org/10.1002/hsr2.70335 10.1002/hsr2.70335 PMC11726644 39807483

[CR17] Crisafulli O, Bottoni G, Lacetera J, Fassio F, Grattarola L, Lavaselli E, Giovanetti G, Tupler R, Negro M, D’Antona G (2024a) Bioimpedance analysis of fat free mass and its subcomponents and relative associations with maximal oxygen consumption in facioscapulohumeral dystrophy. Eur J Appl Physiol. 10.1007/s00421-024-05581-5 39168898 PMC11753324

[CR19] Crisafulli O, Grattarola L, Bottoni G, Lacetera J, Lavaselli E, Beretta-Piccoli M, Tupler R, Soldini E, Antona D, G (2024b) Maximal oxygen consumption is negatively associated with fat mass in facioscapulohumeral dystrophy. Int J Environ Res Public Health 21(8). 10.3390/ijerph21080979 PMC11353994 39200589

[CR20] Crisafulli O, Lacetera J, Bottoni G, Berardinelli A, Grattarola L, Veltroni M, Acquadro S, Negro M, Lavaselli E, D’Antona G (2024c) Case report: A creatine kinase-borg scale values-based approach to tailor physical training in a central core myopathy patient. Front Physiol 15:1404657. 10.3389/fphys.2024.1404657 39108538 PMC11300344

[CR21] Crisafulli O, Sozzi S, Quintiero V, Negro M, Tupler R, Ramat S, Stella GM, Schmid M, D’Antona G (2025b) Interplay between balance, gait kinematic and physical activity level in facioscapulohumeral muscular dystrophy. Sci Rep 15(1):42973. 10.1038/s41598-025-26924-y 41331267 PMC12673088

[CR18] Crisafulli O, Diotti D, Negro M, Lavaselli E, Peters M, Quintiero V, D’Antona G (2025c) Physical exercise in metabolic myopathies at risk of rhabdomyolysis: a feasible approach or an unavoidable hazard? Eur J Appl Physiol 125(11):3039–3064. 10.1007/s00421-025-05922-y 40759836 PMC12528310

[CR22] Cup EH, Pieterse AJ, Broek-Pastoor T, Munneke JM, van Engelen M, Hendricks BG, van der Wilt HT, G. J., Oostendorp RA (2007) Exercise therapy and other types of physical therapy for patients with neuromuscular diseases: a systematic review. Arch Phys Med Rehabil 88(11):1452–1464. 10.1016/j.apmr.2007.07.024 17964887

[CR23] D’Antona G, Brocca L, Pansarasa O, Rinaldi C, Tupler R, Bottinelli R (2007) Structural and functional alterations of muscle fibres in the novel mouse model of facioscapulohumeral muscular dystrophy. J Physiol 584(Pt 3):997–1009. 10.1113/jphysiol.2007.141481 17855756 PMC2277004

[CR24] Dahlqvist JR, Poulsen NS, Østergaard ST, Fornander F, de Stricker Borch J, Danielsen ER, Thomsen C, Vissing J (2020) Evaluation of inflammatory lesions over 2 years in facioscapulohumeral muscular dystrophy. Neurology 95(9):e1211–e1221. 10.1212/wnl.0000000000010155 32611642

[CR25] Deng B, Li H, Chen C, He J, Zhang W, Lin H (2025) Dietary supplement strategies during conditioning training in athletes: a network meta-analysis of peak and mean anaerobic power, VO(2)max, and endurance performance. Food Sci Nutr 13(12):e71243. 10.1002/fsn3.71243 41323837 PMC12663695

[CR26] Eichinger K, Heatwole C, Iyadurai S, King W, Baker L, Heininger S, Bartlett A, Dilek N, Martens WB, McDermott M, Kissel JT, Tawil R, Statland JM (2018) Facioscapulohumeral muscular dystrophy functional composite outcome measure. Muscle Nerve. 10.1002/mus.26088 29381807 PMC6066464

[CR27] Esbjörnsson M, Sylvén C, Holm I, Jansson E (1993) Fast twitch fibres may predict anaerobic performance in both females and males. Int J Sports Med 14(5):257–263. 10.1055/s-2007-1021174 8365833

[CR28] Flanigan KM, Coffeen CM, Sexton L, Stauffer D, Brunner S, Leppert MF (2001) Genetic characterization of a large, historically significant Utah kindred with facioscapulohumeral dystrophy. Neuromuscul Disord 11(6–7):525–529. 10.1016/s0960-8966(01)00201-2 11525880

[CR29] Foster-Schubert KE, Alfano CM, Duggan CR, Xiao L, Campbell KL, Kong A, Bain CE, Wang CY, Blackburn GL, McTiernan A (2012) Effect of diet and exercise, alone or combined, on weight and body composition in overweight-to-obese postmenopausal women. Obes (Silver Spring) 20(8):1628–1638. 10.1038/oby.2011.76 PMC3406229 21494229

[CR30] Fowler WM Jr. (1984) Importance of overwork weakness. Muscle Nerve 7(6):496–499 6543905

[CR31] Fowler WM Jr., Taylor M (1982) Rehabilitation management of muscular dystrophy and related disorders: I. The role of exercise. Arch Phys Med Rehabil 63(7):319–321 7092533

[CR32] Gianola S, Castellini G, Pecoraro V, Monticone M, Banfi G, Moja L (2020) Effect of muscular exercise on patients with muscular dystrophy: a systematic review and meta-analysis of the literature. Front Neurol 11:958. 10.3389/fneur.2020.00958 33281695 PMC7688624

[CR33] Glynn EL, Fry CS, Drummond MJ, Timmerman KL, Dhanani S, Volpi E, Rasmussen BB (2010) Excess leucine intake enhances muscle anabolic signaling but not net protein anabolism in young men and women. J Nutr 140(11):1970–1976. 10.3945/jn.110.127647 20844186 PMC2955876

[CR34] Guien C, Blandin G, Lahaut P, Sanson B, Nehal K, Rabarimeriarijaona S, Bernard R, Lévy N, Sacconi S, Béroud C (2018) The French National Registry of patients with Facioscapulohumeral muscular dystrophy. Orphanet J Rare Dis 13(1):218. 10.1186/s13023-018-0960-x 30514324 PMC6280451

[CR35] Gunnarsson TP, Bangsbo J (2012) The 10-20-30 training concept improves performance and health profile in moderately trained runners. J Appl Physiol (1985) 113(1):16–24. 10.1152/japplphysiol.00334.2012 22556401

[CR36] Hord JM, Burns S, Willer T, Goddeeris MM, Venzke D, Campbell KP (2025) Sarcolemma resilience and skeletal muscle health require O-mannosylation of dystroglycan. Skelet Muscle 15(1):1. 10.1186/s13395-024-00370-2 39789642 PMC11715199

[CR37] Horwath O, Montiel-Rojas D, Ponsot E, Féasson L, Kadi F (2025) Increased muscle satellite cell content and preserved telomere length in response to combined exercise training in patients with FSHD. J Physiol 603(5):1057–1069. 10.1113/jp287033 39891610 PMC11870062

[CR38] Hung CH, Su CH, Wang D (2025) The role of high-intensity interval training (HIIT) in neuromuscular adaptations: implications for strength and power development-a review. Life (Basel) 15(4). 10.3390/life15040657 PMC12028608 40283211

[CR39] Iannetta D, Inglis EC, Mattu AT, Fontana FY, Pogliaghi S, Keir DA, Murias JM (2020) A critical evaluation of current methods for exercise prescription in women and men. Med Sci Sports Exerc 52(2):466–473. 10.1249/mss.0000000000002147 31479001

[CR40] Jäger R, Kerksick CM, Campbell BI, Cribb PJ, Wells SD, Skwiat TM, Purpura M, Ziegenfuss TN, Ferrando AA, Arent SM, Smith-Ryan AE, Stout JR, Arciero PJ, Ormsbee MJ, Taylor LW, Wilborn CD, Kalman DS, Kreider RB, Willoughby DS, Antonio J (2017) International Society of Sports Nutrition Position Stand: protein and exercise. J Int Soc Sports Nutr 14:20. 10.1186/s12970-017-0177-8 28642676 PMC5477153

[CR41] Janssen B, Voet N, Geurts A, van Engelen B, Heerschap A (2016) Quantitative MRI reveals decelerated fatty infiltration in muscles of active FSHD patients. Neurology 86(18):1700–1707. 10.1212/wnl.0000000000002640 27037227

[CR42] Johnson EW, Braddom R (1971) Over-work weakness in facioscapulohuumeral muscular dystrophy. Arch Phys Med Rehabil 52(7):333–336 5565885

[CR43] Lak M, Bagheri R, Ghobadi H, Campbell B, Wong A, Shahrbaf A, Shariatzadeh M, Dutheil F (2024) Timing matters? The effects of two different timing of high protein diets on body composition, muscular performance, and biochemical markers in resistance-trained males. Front Nutr 11:1397090. 10.3389/fnut.2024.1397090 38846541 PMC11156191

[CR44] Lamperti C, Fabbri G, Vercelli L, D’Amico R, Frusciante R, Bonifazi E, Fiorillo C, Borsato C, Cao M, Servida M, Greco F, Di Leo R, Volpi L, Manzoli C, Cudia P, Pastorello E, Ricciardi L, Siciliano G, Galluzzi G, Tupler R (2010) A standardized clinical evaluation of patients affected by facioscapulohumeral muscular dystrophy: The FSHD clinical score. Muscle Nerve 42(2):213–217. 10.1002/mus.21671 20544930

[CR45] Lassche S, Stienen GJ, Irving TC, van der Maarel SM, Voermans NC, Padberg GW, Granzier H, van Engelen BG, Ottenheijm CA (2013) Sarcomeric dysfunction contributes to muscle weakness in facioscapulohumeral muscular dystrophy. Neurology 80(8):733–737. 10.1212/WNL.0b013e318282513b 23365058 PMC3589299

[CR46] Leung DG, Carrino JA, Wagner KR, Jacobs MA (2015) Whole-body magnetic resonance imaging evaluation of facioscapulohumeral muscular dystrophy. Muscle Nerve 52(4):512–520. 10.1002/mus.24569 25641525 PMC4504833

[CR47] MacInnis MJ, Gibala MJ (2017) Physiological adaptations to interval training and the role of exercise intensity. J Physiol 595(9):2915–2930. 10.1113/JP273196 27748956 PMC5407969

[CR48] Mah JK, Korngut L, Dykeman J, Day L, Pringsheim T, Jette N (2014) A systematic review and meta-analysis on the epidemiology of Duchenne and Becker muscular dystrophy. Neuromuscul Disord 24(6):482–491. 10.1016/j.nmd.2014.03.008 24780148

[CR49] Mann T, Lamberts RP, Lambert MI (2013) Methods of prescribing relative exercise intensity: physiological and practical considerations. Sports Med 43(7):613–625. 10.1007/s40279-013-0045-x 23620244

[CR50] McDonald CM (2010) Physical activity and exercise in FSHD: a physician’s and a patients perspectives https://www.fshdsociety.org/wp-content/uploads/2019/08/FSHSocietyPatientIPRN2010_McDonald-min.pdf

[CR51] Mellion ML, Widholm P, Karlsson M, Ahlgren A, Tawil R, Wagner KR, Statland JM, Wang L, Shieh PB, van Engelen BGM, Kools J, Ronco L, Odueyungbo A, Jiang J, Han JJ, Hatch M, Towles J, Leinhard OD, Cadavid D (2022) Quantitative muscle analysis in FSHD using whole-body fat-referenced mri: composite scores for longitudinal and cross-sectional analysis. Neurology 99(9):e877–e889. 10.1212/wnl.0000000000200757 35750498

[CR52] Naderi A, Rothschild JA, Santos HO, Hamidvand A, Koozehchian MS, Ghazzagh A, Berjisian E, Podlogar T (2025) Nutritional strategies to improve post-exercise recovery and subsequent exercise performance: a narrative review. Sports Med 55(7):1559–1577. 10.1007/s40279-025-02213-6 40221559 PMC12297025

[CR53] Nigro V, Piluso G (2015) Spectrum of muscular dystrophies associated with sarcolemmal-protein genetic defects. Biochim Biophys Acta 1852(4):585–593. 10.1016/j.bbadis.2014.07.023 25086336

[CR54] Olsen DB, Ørngreen MC, Vissing J (2005) Aerobic training improves exercise performance in facioscapulohumeral muscular dystrophy. Neurology 64(6):1064–1066. 10.1212/01.Wnl.0000150584.45055.27 15781829

[CR55] Ozawa E, Nishino I, Nonaka I (2001) Sarcolemmopathy: muscular dystrophies with cell membrane defects. Brain Pathol 11(2):218–230. 10.1111/j.1750-3639.2001.tb00394.x 11303797 PMC8098542

[CR56] Pang MY, Eng JJ, Dawson AS, Gylfadóttir S (2006) The use of aerobic exercise training in improving aerobic capacity in individuals with stroke: a meta-analysis. Clin Rehabil 20(2):97–111. 10.1191/0269215506cr926oa 16541930 PMC3167867

[CR57] Pasotti S, Magnani B, Longa E, Giovanetti G, Rossi A, Berardinelli A, Tupler R, D’Antona G (2014) An integrated approach in a case of facioscapulohumeral dystrophy. BMC Musculoskelet Disord 15:155. 10.1186/1471-2474-15-155 24886582 PMC4032568

[CR58] Pini J, Martinuzzi E, Dhifallah S, Slioui A, Puma A, Villa L, Cavalli M, Ezaru A, Garcia J, Gambella M, Torre F, Pavan LJ, Glaichenhaus N, Sacconi S (2026) Interleukin-6 as a key biomarker in facioscapulohumeral dystrophy: evidence from longitudinal analyses. Ann Clin Transl Neurol 13(2):310–323. 10.1002/acn3.70210 41058127 PMC12883698

[CR59] Prieur-Blanc N, Cotinat M, Vansteenkiste S, de Bovis Milhe V, Viton JM, Attarian S, Bensoussan L (2024) Fitness and walking outcomes following aerobic and lower extremity strength training in facioscapulohumeral dystrophy: a case series. Int J Rehabil Res 47(1):41–45. 10.1097/mrr.0000000000000614 38323889

[CR61] Quintiero V, Crisafulli O, Lacetera J, Bottoni G, Negro M, Tupler R, Lavaselli E, Antona D (2025a) Effects of a combined nutritional and physical training program approach in a case of facioscapulohumeral dystrophy: a one-year follow-up. Case Rep Med 2025:7873892. 10.1155/carm/7873892 PMC12436011 40959201

[CR60] Quintiero V, Crisafulli O, Diotti D, Tupler R, Negro M, Lavaselli E, Antona D, G (2025b) State-of-the-art and future challenges for nutritional interventions in facioscapulohumeral dystrophy: a narrative review. Nutrients 17(6). 10.3390/nu17061056 PMC11944979 40292463

[CR62] Ricci G, Ruggiero L, Vercelli L, Sera F, Nikolic A, Govi M, Mele F, Daolio J, Angelini C, Antonini G, Berardinelli A, Bucci E, Cao M, D’Amico MC, D’Angelo G, Di Muzio A, Filosto M, Maggi L, Moggio M, Tupler R (2016) A novel clinical tool to classify facioscapulohumeral muscular dystrophy phenotypes. J Neurol 263(6):1204–1214. 10.1007/s00415-016-8123-2 27126453 PMC4893383

[CR63] Rijken NH, van Engelen BG, de Rooy JW, Geurts AC, Weerdesteyn V (2014) Trunk muscle involvement is most critical for the loss of balance control in patients with facioscapulohumeral muscular dystrophy. Clin Biomech (Bristol) 29(8):855–860. 10.1016/j.clinbiomech.2014.07.008 25156185

[CR64] Rogeri PS, Zanella R Jr, Martins GL, Garcia MDA, Leite G, Lugaresi R, Gasparini SO, Sperandio GA, Ferreira LHB, Souza-Junior TP, Lancha AH Jr (2021) Strategies to prevent sarcopenia in the aging process: role of protein intake and exercise. Nutrients 14(1). 10.3390/nu14010052 PMC8746908 35010928

[CR102] Salort-Campana E, Fatehi F, Beloribi-Djefaflia S, Roche S, Nguyen K, Bernard R, Cintas P, Solé G, Bouhour F, Ollagnon E, Sacconi S, Echaniz-Laguna A, Kuntzer T, Levy N, Magdinier F, Attarian S (2020) Type 1 FSHD with 6–10 repeated units: factors underlying severity in index cases and disease penetrance in their relatives attention. Int J Mol Sci 21(6). https://doi.org/10.3390/ijms21062221 10.3390/ijms21062221 PMC7139460 32210100

[CR65] Schätzl T, Kaiser L, Deigner HP (2021) Facioscapulohumeral muscular dystrophy: genetics, gene activation and downstream signalling with regard to recent therapeutic approaches: an update. Orphanet J Rare Dis 16(1):129. 10.1186/s13023-021-01760-1 33712050 PMC7953708

[CR66] Schipper K, Bakker M, Abma T (2017) Fatigue in facioscapulohumeral muscular dystrophy: a qualitative study of people’s experiences. Disabil Rehabil 39(18):1840–1846. 10.1080/09638288.2016.1212109 27762634

[CR67] Skalsky AJ, Abresch RT, Han JJ, Shin CS, McDonald CM (2008) The relationship between regional body composition and quantitative strength in facioscapulohumeral muscular dystrophy (FSHD). Neuromuscul Disord 18(11):873–880. 10.1016/j.nmd.2008.07.005 18818077

[CR68] Vera KA, McConville M, Glazos A, Stokes W, Kyba M, Keller-Ross M (2022) Exercise intolerance in facioscapulohumeral muscular dystrophy. Med Sci Sports Exerc 54(6):887–895. 10.1249/mss.0000000000002882 35195100 PMC9117420

[CR69] Vincenten SCC, Teeselink S, Mul K, Heskamp L, Kan HE, Heerschap A, Cameron D, Tasca G, Leung DG, Voermans NC, van Engelen BGM, van Alfen N (2025) Muscle imaging in facioscapulohumeral muscular dystrophy research: A scoping review and expert recommendations. Neuromuscul Disord 47:105274. 10.1016/j.nmd.2025.105274 39884029

[CR71] Voet NBM (2019) Exercise in neuromuscular disorders: a promising intervention. Acta Myol 38(4):207–214 31970319 PMC6955632

[CR70] Voet N, Bleijenberg G, Hendriks J, de Groot I, Padberg G, van Engelen B, Geurts A (2014) Both aerobic exercise and cognitive-behavioral therapy reduce chronic fatigue in FSHD: an RCT. Neurology 83(21):1914–1922. 10.1212/wnl.0000000000001008 25339206

[CR72] Voet NBM, Saris CGJ, Thijssen DHJ, Bastiaans V, Sluijs DE, Janssen M (2022) Surface electromyography thresholds as a measure for performance fatigability during incremental cycling in patients with neuromuscular disorders. Front Physiol 13:821584. 10.3389/fphys.2022.821584 35370798 PMC8969223

[CR73] Witard OC, Hearris M, Morgan PT (2025) Protein nutrition for endurance athletes: a metabolic focus on promoting recovery and training adaptation. Sports Med 55(6):1361–1376. 10.1007/s40279-025-02203-8 40117058 PMC12152099

[CR74] Wolpern AE, Burgos DJ, Janot JM, Dalleck LC (2015) Is a threshold-based model a superior method to the relative percent concept for establishing individual exercise intensity? A randomized controlled trial. BMC Sports Sci Med Rehabil 7:16. 10.1186/s13102-015-0011-z 26146564 PMC4491229

[CR75] Xie Y, Gu Y, Li Z, He B, Zhang L (2024) Effects of different exercises combined with different dietary interventions on body composition: a systematic review and network meta-analysis. Nutrients 16(17). 10.3390/nu16173007 PMC11397086 39275322

