# Supplementary material for: Physical exercise in facioscapulohumeral muscular dystrophy: state of the art and future challenges beyond common misconceptions
Source: Eur J Appl Physiol. 2026 Jul 2;126(9):4651–72. doi: 10.1007/s00421-026-06338-y (PMC13541994; doi:10.1007/s00421-026-06338-y)
Supplement: Supplementary file 1 [file 421_2026_6338_MOESM1_ESM.docx]

Supplementary Table 1. Description of the studies adopting aerobic, anaerobic, and combined aerobic-anaerobic exercise training in FSHD patients

| **Author(s) and years** | **Subjects** | **Exercise/nutritional intervention and duration** | **Between-group changes** | **Within-group changes** |
| --- | --- | --- | --- | --- |
| ***Aerobic exercise training*** | | | | |
| *Randomised controlled trials* | | | | |
| (Voet et al., 2014) | 57 FSHD* Type 1 patients subdivided into:  a) AET (n= 20; 8F; median age 59 y (21-68); Ricci Score 3.0 (0.5-4.0); duration of illness median 13.0 y (1.0-42.0); ClS-fatigue 41 (35-54))  b) CBT (n= 13; 5F; median age 49 y (24-69); Ricci Score 2.0 (1.0-4.0); duration of illness median 7.0 y (0.0-40.0); ClS-fatigue 43 (35-56))  c) UC (n= 24; 7F; median age 52 y (20-79); Ricci Score 3.0 (1.5-4.0); duration of illness median 16.7 y (1.0-49.0); ClS-fatigue 42 (35-51)) | **Aerobic training:** cycle-ergometer, 3 sessions per week for 16 weeks for a duration of 30 minutes, with intensity gradually increased from 50% to 65% of HRR.  **Cognitive-behavioural therapy,** a 50-minute lessons directed at insufficient coping with the disease.  **Usual care**  No treatment or occasional (conventional) physical therapy. | ***Pre- vs post-intervention (16^th^ weeks)***  *V̇O_2_peak (L/min):*  AET = UC, (+0.1)  CBT = UC, (+0.2)  *6MWT (m):*  AET = UC, (+18)  CBT = UC, (-9)  *Daily activity (5-min averaged body accelerations):*  AET > UC, (+4.6, p<0.05)  CBT > UC, (+5.6, p<0.05)  *CIS-fatigue (pts):*  AET > UC, (-9.1, p<0.05)  CBT > UC, (-13.3, p<0.05)  *MVIC for the quadriceps (kg):*  AET > UC, (+2.1, p<0.05)  CBT = UC, (-0.2)  ***Pre- vs post-follow up (28^th^ week)***  *V̇O_2_peak (L/min):*  AET = UC, (+0.2)  CBT = UC, (+0.3)  *6MWT (m):*  AET = UC, (+15)  CBT = UC, (-12)  *Daily activity (5-min averaged body accelerations):*  AET > UC, (+5.5, p<0.05)  CBT > UC, (+7.1, p<0.05)  *CIS-fatigue (pts):*  AET > UC, (-8.2, p<0.05)  CBT > UC, (-10.2, p<0.05)  *MVIC for the quadriceps (kg):*  AET = UC, (+2.3)  CBT = UC (+0.9) | ***Pre- vs post-intervention (16^th^ week) vs follow-up (28^th^ week), median (range)):***  *V̇O_2_peak (L/min):*  AET, pre: 2.50 (1.40-3.30) vs post: 2.35 (1.70-3.75) vs follow-up: 2.70 (2.10-3.30)  CBT, pre: 2.75 (1.60-5.15) vs post: 2.73 (1.90-4.60) vs follow-up: 2.80 (1.90-5.15)  UC, pre: 2.65 (1.20-5.65) vs post: 2.50 (1.20-4.30) vs follow-up: 2.70 (1.50-3.80)  *6MWT (m):*  AET, pre: 388 (136-630) vs post: 420 (159-605) vs follow-up: 450 (157-622)  CBT, pre: 457 (178-650) vs post: 440 (210-654) vs follow-up: 459 (120-733)  UC, pre: 436 (80-708) vs post: 430 (90-800) vs follow-up: 461 (86-832)  *Daily activity (5-min averaged body accelerations):*  AET, pre: 32 (6-68) vs post: 38 (15-65) vs follow-up: 32 (1-60)  CBT, pre: 43 (21-69) vs post: 43 (17-73) vs follow-up: 41 (17-65)  UC, pre: 38 (19-74) vs post: 36 (10-72) vs follow-up: 32 (12-70)  *CIS-fatigue (pts):*  AET, pre: 41 (35-54) vs post: 38 (18-53) vs follow-up: 39 (11-53)  CBT, pre: 43 (35-56) vs post: 24 (12-56) vs follow-up: 28 (16-56)  UC, pre: 42 (35-51) vs post: 42 (32-56) vs follow-up: 42 (35-56)  *MVIC for the quadriceps (kg):*  AET, pre: 21.0 (10.0-42.7) vs post: 26.0 (9.1-35.7) vs follow-up: 24.8 (5.6-47.4)  CBT, pre: 21.6 (9.2-40.7) vs post: 20.1 (9.6-33.1) vs follow-up: 20.7 (9.6-39.1)  UC, pre: 19.7 (1.9-40.3) vs post: 15.0 (2.0-39.5) vs follow-up: 17.7 (0.9-58.0) |
| (Janssen et al., 2016) | 31 FSHD* Type 1 patients subdivided into:  a) AET (n= 9; 4F; mean age 56±15 y; Ricci score: 3.1±1.1)  b) CBT (n= 9; 3F; mean age 53±12 y; Ricci Score: 2.3±1.0)  c) UC (n= 13; 3F; mean age 53±15 y; Ricci score: 3.0±0.9) | **Aerobic training:** cycle-ergometer, 3 sessions per week for 16 weeks for a duration of 30 minutes, with intensity gradually increased from 50% to 65% of HRR.  **Cognitive-behavioural therapy,** a 50-minute lessons directed at insufficient coping with the disease, plus a maximum of 2 60-minute sessions of walking or cycling per day.  **Usual care**  No treatment or occasional (conventional) physical therapy. | ***Pre- vs post-intervention (16^th^ week)***  *Progression of fatty infiltration:*  AET < UC (p=0.03)  CBT < UC (p=0.0015) | ***Pre- vs post-intervention (16^th^ week)***  *Progression of fatty infiltration:*  UC: +6.7%/years  AET: +2.9%/years  CBT: +1.7%/years |
| ***Randomised controlled trials plus nutritional intervention*** | | | | |
| (Andersen, Prahm, et al., 2015) | 35 FSHD* 1 Patients subdivided into:  a) AET+SUP (n= 13; 5F; mean 42.6 y (24-55); FSHD Clinical Score: 5.2 (3-7)),  b) AET+PLA (n= 13; 6F; mean 45.7 y (22-63); FSHD Clinical Score: 6.2 (1-11))  c) CG (n= 9; 4F; mean 51.3 y (24-65); FSHD Clinical Score: 5.9 (2-9)) | **Aerobic training, cycle-ergometer,** 3 sessions per week for 12 weeks, with sessions building up from 15 minutes (1^st^ week) to 30 minutes (3^rd^ week and thereafter), with an intensity of 70% V̇O_2_max.  **Nutritional intervention**  Protein-Carbohydrate supplement: 163 kcal (23 g whey protein, 17 g carbohydrate, 0 g fat).  Placebo: (0 kcal)  **Control group**  No nutritional and physical intervention | ***Pre- vs post-intervention (12^th^ week)***  AET+SUP did not improve fitness, workload, or walking speed further than AET+PLA. | ***Pre- vs post-intervention (12^th^ week)***  *V̇O_2_max (ml·min⁻¹·kg⁻¹):*  **↑** AET+(SUP & PLA) (+9.5%, p<0.002)  = CG  *Wmax (W):*  **↑** AET+(SUP & PLA) (17.7%, p<0.001)  = CG  *Walking speed (km/h):*  **↑** AET+(SUP & PLA) (+7.4%, p<0.001)  = CG  *Stand-balance test, stair test at self- and fast pace, chair test at fast pace (s):*  = AET+(SUP & PLA)  = CG  *Daily activity (steps/day):*  = AET+(SUP & PLA)  = CG  *Plasma CK (U/L):*  = AET+(SUP & PLA)  *Level of pain and fatigue:*  = AET+(SUP & PLA)  *MVIC (N):*  = AET+(SUP & PLA)  = CG |
| ***Non-randomised controlled trial*** | | | | |
| (Olsen et al., 2005) | 8 FSHD* AET (n= 8; 4F; mean age: 36 y (18-55))  7 HC AET (n= 7; 3F; mean age: 49 y (35-60)) | **Aerobic training**, cycle-ergometer, the number of weekly sessions increased during the first 4 weeks until reaching five times/week, totalising 44 sessions in 12 weeks, for a duration of 35 minutes, with an intensity of 65% V̇O_2_max. | - | ***Pre- vs post-intervention (12^th^ week):***  *V̇O_2_max (ml·min⁻¹·kg⁻¹):*  ↑ FSHD AET (+16 ± 3%, p<0.002)  ↑ HC AET (+13 ± 3%, p<0.02)  *Wmax (W):*  ↑ FSHD AET (+17 ± 4%, p<0.002)  ↑ HC AET (+13 ± 6%, p<0.006)  *Type 1 fibres distribution (%):*  = FSHD AET  = HC AET  *Type 2 fibres distribution (%):*  = FSHD AET  = HC AET  *Muscle fibre CSA (μm^2^):*  = FSHD AET  = HC AET  *Capillary density (cap/mm^2^):*  = FSHD AET  = HC AET  *Blood plasma CK (U/L, pre- vs 6^th^ week):*  = FSHD AET (Pre: 514 ± 179, Post: 327 ± 152) |
| **Anaerobic exercise training** | | | | |
| ***Randomized controlled trials*** | | | | |
| (Andersen et al., 2017) | 12 FSHD* 1 Patients subdivided into:  a) Supervised HIT (n= 6; 2F; mean 53±15 y (26-67); Clinical Score: 6.3±4.3 (1-11))  b) UC (n= 6; 1F; mean 46±9 y (32-59); Clinical Score: 7.5±2.1 (5-11))  6 HC inserted into:  c) HC Supervised HIT (n= 6; 2F; mean 52±14 (27-64)) | **High-intensity interval training:** cycle-ergometer, 3 sessions per week for 8 weeks (supervised) + 8 weeks (unsupervised) for a duration of 21 minutes, in which two sets of five consecutive 1-min HIT bouts (30 s at low intensity, 20 s at middle-high intensity, and 10 s of all-out, maximal intensity) were performed.  **Usual care group**  Not specified | - | ***Pre vs post-intervention (8^th^ week)***  *V̇O_2_max (ml·min⁻¹·kg⁻¹):*  **↑** FSHD Supervised HIT (+16±4%, +3.5 ± 0.9, p<0.01)  = FSHD UC  = HC Supervised HIT  *Wmax (W):*  **↑** FSHD Supervised HIT (+17 ± 5%, p=0.03)  = FSHD UC  **↑** HC Supervised HIT (+8 ± 7%, p=0.03)  *MVIC (N):*  = FSHD Supervised HIT  = FSHD UC  = HC Supervised HIT  *6MWT (m):*  = FSHD Supervised HIT  **↑** FSHD UC (+21 ± 16 m, p<0.03)  = HC Supervised HIT  *5STS (s):*  = FSHD Supervised HIT  = FSHD UC  = HC Supervised HIT  *Daily activity (step/day):*  = FSHD Supervised HIT  = FSHD UC  = HC Supervised HIT  ***Pre- vs during intervention:***  *Plasma CK (*U/l)*:*  = FSHD Supervised HIT (67–600 vs 71–700) |
| **Combined aerobic-anaerobic exercise training** | | | | |
| ***Randomised controlled trials*** | | | | |
| (Bankolé et al., 2016) | 16 FSHD* 1 type patients subdivided into:  a) TG (n= 8; 3F; mean age 40±13 y)  b) CG (n= 8; 1F; mean age 41±9 y)  Afterwards, CG was inserted in the intervention as:  c) TCG | **Combined aerobic/strength exercise,** cycle-ergometer, 3 sessions per week for 24 weeks, for a duration of 35 minutes, of which two sessions consisted of 25-min constant moderate intensity (60% of MAP), followed by 5 sets of 10 near-maximal revolutions, while the third session was composed by five 1-min intervals at 80% MAP. | - | ***Pre- vs post-intervention (24^th^ week):***  *V̇O_2_peak (ml·min⁻¹·kg⁻¹):*  **↑** TG (Pre: 27.3 ± 12.2, Post: 33.6 ± 12.4, p< 0.001)  = CG  **↑** TCG (+17%, p< 0.001)  *MAP (W):*  **↑** TG (Pre: 125 ± 79, Post: 170 ± 87, p< 0.001)  = CG  **↑** TCG (+30%, p< 0.001)  *MVIC (N^.^m):*  **↑** TG (Pre: 125 ± 47, Post: 139 ± 47, p<0.01)  = CG  = TCG  *Muscle endurance (number of repetitions):*  **↑** TG (Pre: 56 ± 7, Post: 66 ± 14, p<0.05)  = CG **↑** TCG (+11%, p<0.01)  *6MWT (m):*  **↑** TG (Pre: 508 ± 129, Post: 570 ± 130, p<0.001)  = CG  **↑** TCG (+13%, p<0.01)  *FSS (pts):*  **↓** TG (Pre: 44 ± 8, Post: 34 ± 15, p<0.01)  = CG  **↓** TCG (-22%, p=0.001)  *SF-36 (pts):*  = TG  = CG  = TCG  *Whole muscle fibre CSA (μm^2^):*  **↑** TG (Pre: 5392 ± 1165, Post: 7214 ± 2247, p<0.05)  = CG  *Citrate synthase activity (μmol μmin^–1^·μg^–1^· dry weight):*  **↑** FSHD TG (Pre: 37.0 ± 15.3, Post: 51.3 ± 14.1, p=0.005)  = CG  *Plasma CK (IU·L^-1^):*  = FSHD TG (<1000 at all time points)  = TCG (<1000 at all time points) |
| (Horwath et al., 2025) | 16 FSHD* Type 1 patients subdivided into:  a) TG (n= 8; 3F; 40±13 y)  b) CG (n= 8; 1F; 44±10 y) | **Combined aerobic/strength exercise,** cycle-ergometer, 3 sessions per week for 24 weeks, for a duration of 35 minutes, of which two sessions consisted of 25-min constant moderate intensity (60% of MAP), followed by 5 sets of 10 near-maximal revolutions, while the third session was composed by five 1-min intervals at 80% MAP. | - | *Proportion of fibre with CSAs between 3000-4000 μm^2^ in slow fibre:*  **↓** TG (p=0.022)  = CG  *Proportion of fibre with CSAs between 3000-4000 μm^2^ in fast fibre:*  **↓** TG (p=0.020)  = CG  *Proportion of fibre with CSAs between 5000-6000 μm^2^ in slow fibre:*  = TG  = CG  *Proportion of fibre with CSAs between 5000-6000 μm^2^ in fast fibre:*  **↑** TG (p=0.008)  = CG  *Satellite cell pool expansion in fast muscle fibres (Pax7+ cells per type IIA fiber):*  **↑** TG (+75%, p=0.015)  = CG |
| ***Non-randomised controlled trial*** | | | | |
| (Prieur-Blanc et al., 2024) | 10 FSHD* patients (n= 10; 7F, mean age: 50.7±15.2 y) | Rehabilitation program, composed of aerobic, strength, and standard occupational therapy of 5 hospital-based rehabilitation sessions for 4 week.  *Aerobic component*: cycle-ergometer for 30 minutes at an intensity corresponding to the first ventilatory threshold.  *Strength training*: isokinetic device for a total of 10 sets at different angular velocities at 40% of the maximum concentric strength and increasing by 10% every five sessions, with a rest period of 45 seconds.  *Conventional therapy*: 1h of strength training and articular mobilisation of the upper limbs | - | ***Pre- vs post-intervention (4^th^ week):***  ↑ *V̇O_2_max* (Pre: 20.0 ± 4.0, Post: 22.1 ± 5.1 ml·min⁻¹·kg⁻¹, p=0.022)  ↑ *MAP* (Pre: 75.0 ± 26.9, Post: 83.8 ± 32.0 W, p=0.044)  ↑ *6MWT* (Pre: 355.3 ± 94.46, Post: 447.4 ± 89.30 m, p=0.016)  ↑ *10mWT* (Pre: 1.18 ± 0.25, Post: 1.46 ± 0.29 m/s, p=0.002)  ↑ *Knee flexors at 60°/s* (Pre: 0.938 ± 0.58, Post: 0.969 ± 0.55 Nm/Kg, p=0.031)  = *Knee flexors at 120°/s and 180°/s*  = *Knee extensors at 60°/s, 120°/s, and 180°/s*  **↓** *Visual number scale physical fatigue* (Pre: 39 ± 3, Post: 5 ± 1.8 pts, p=0.016)  **↓** *Insomnia Severity Index* (Pre: 8.3 ± 7.5, Post: 3.5 ± 4,95 pts, p=0.022) |
| ***Case study*** | | | | |
| (Calisgan et al., 2019) | 1 FSHD* (n= 1; F; 26 y) | Combined exercise training, composed of stretching, strength, endurance, and aerobic exercises, for 3 days per week for 4 months, plus kinesiotape once per week. | - | ***Pre- vs post-intervention (24^th^ week):***  ↑ *ROM right side* (7 out of 10 movements)  ↑ *ROM left side* (6 out of 10 movements)  ↑ *Muscle strength, right side* (16 out of 23 muscle functions)  ↑ *Muscle strength, left side* (15 out of 23 muscle functions)  ↑ *6MWT* (Pre: 300, Post: 360 m)  **↓** *10mWT* (Pre: 12, Post: 8.6 s)  **↓** *Climbing stairs with assistance* (Pre: 12, Post: 10 s)  **↓** *Climbing stairs without assistance* (Pre: 18, Post: 13.07 s)  **↓** *Stepping downstairs with assistance* (Pre: 7, Post: 5.6 s)  **↓** *Stepping down without assistance* (Pre: 18, Post: 5.6 s)  **↓** *FSS* (Pre: 6.22, Post: 3.88 pts) |
| ***Case study plus nutritional intervention*** | | | | |
| (Pasotti et al., 2014) | 1 FSHD* (n= 1; F; 43 y; FSHD score 12) | Combined endurance and strength training, plus nutritional intervention  *Aerobic training*, arm cycle-ergometer for 2 sessions/week for 24 weeks, starting from 10 min and progressively building up to 20 min from week 13 at an intensity of 130 beats per minute.  *Strength training*, with an elastic band for 2 sessions/week for 24 weeks, for 2 sets of 8 repetitions with 5 min rest between series at 50- 60% of MVC.  *Respiratory muscle training:* 4 times/week for 2 min (50% vital capacity at 20 min^−1^) of isocapnic hyperpnea.  *Nutritional intervention*  Supplementation was provided with BCAA, creatine monohydrate and CLA | - | ***Pre- vs post-intervention (24^th^ week):***  **↓** *V̇O_2_(7.5Watt)* (Pre: 11.76, Post: 7.9 ml·kg⁻¹·min⁻¹)  **↓** *VE* (Pre: 30, Post: 20 L/min)  ↑ *Shoulder abductors strength*  Left, Pre: 16.7, Post: 38.2 N;  Right, Pre: 21,6, Post: 39.2 N  **↓**/↑ *Elbow flexors strength*  Left, Pre: 16.7, Post 23.5 N  Right, Pre: 25.5, Post 20.6 N  **↓**/↑ *Elbow extensors strength*  Left, Pre: 12.7, Post: 10.7 N  Right, Pre: 6.9, Post: 11.7 N  = *REE* (Pre: 1358, Post: 1332 kcal/day)  **↓** *Body mass* (Pre: 71.4, Post: 62 kg)  ↓ *FFM* (Pre: 64.2, Post: 72.1 %)  ↓ *FM* (Pre: 36.2, Post: 28.1 %)  = *PFTs* |
| (Quintiero et al., 2025) | 1 FSHD* (n= 1, M, 19 y; subcategory A3) | Combined aerobic-anaerobic exercise  *Aerobic training*, cycle-ergometry, one session/week for 52 weeks for 45 minutes, with an intensity between 40% and 50% of the theoretical HRmax.  *Resistance training*, two sessions/week for 52 weeks, for 60 minutes, performed as 2-4 sets of 10-15 repetitions, with between-sets recovery of 1min and 30s.  *Supplementation*  11 g/day of essential amino acid (Aminotrofic-NE). | - | ***Pre- vs post-intervention (52^nd^ week):***  **↓** *CIS20R* (Pre: 18, Post: 16 pts, −11.1%)  ↑ *FACIT-F* (Pre: 46, Post: 48 pts, +4.3%)  ↑ *Body weight* (Pre: 75.8, Post: 80.6 kg, +6.3%)  ↑ *FFM* (Pre: 66.9, Post: 73.8 kg, +10.3%)  ↑ *BCM* (Pre: 41.2, Post: 44.5 kg, +8.0%)  **↓** *FM* (Pre: 8.9, Post: 6.8 kg, 23.6%)  **↓** *Blood glucose level* (Pre: 6.2, Post: 4.6 mmol/L, −25.8%)  **↓** *Blood insulin level* (Pre: 10.2, Post: 6.1 μUI/mL, −40.2%)  ↑ *RMR* (Pre: 1762, Post: 2071 kcal/day, +17.5%) |

Term legends: *AET* aerobic exercise training; *AET+SUP* aerobic training groups plus supplementation; *AET+PLA* aerobic training groups plus placebo; *AET+(SUP* *& PLA)* aerobic training groups plus supplementation and placebo; *CBT* cognitive-behavioural therapy; *CG* control group; *BCAA* branched-chain amino acids; *BCM* body cell mass; *ClS-fatigue* Checklist Individual Strength – Fatigue; *CLA* conjugated linoleic acid; *CK* creatin kinase; *CSA* cross-sectional area; f females; *FACIT-F* Functional Assessment of Chronic Illness Therapy – Fatigue; *FFM* fat-free mass; *FM* fat mass; *FSHD* facioscapulohumeral muscle dystrophy; *FSS* fatigue severity scale; *HC* healthy controls; *HIT* high-intensity training; *HRmax* heart rate maximum; n number; *HRR* heart rate reserve; *MAP* maximal aerobic power; *MVC* maximal voluntary contraction; *MVIC* maximal voluntary isometric contraction; *p* p-value; *PFT* pulmonary function tests; *Pax7+* paired box protein 7–positive cells; *Pre* pre-intervention; *Post* post-intervention; *REE* resting energy expenditure; *RMR* resting metabolic rate; *ROM* range of motion; *SF-36* short-form health survey; *VE* ventilatory equivalent; *TCG* training control group; *TG* training group; *UC* usual care; *V̇O_2_* volume of oxygen; *V̇O_2_max* maximal oxygen uptake; *V̇O_2_peak* peak oxygen uptake; *vs* versus: *y* years; *6MWT* six-minute walking test; *10* *mWT* ten-meter walking test; *Wmax* watt maximum; *y* years; *5STS* 5-time sit-to-stand test.

Measurement units: *cap/mm^2^* capillaries per square millimetre; *g* grams; *kcal/day* kilocalorie per day; *kg* kilograms, km/*h* kilometres per hour; *IU·L^-1^* international units per litre; *L/min* litres per minute; *m* meters; *min* minutes; *ml·min⁻¹·kg⁻¹* millilitres per minute per kilogram; *μm^2^* square micrometres; *μUI* micro-international units; *μmol μmin^–1^·μg^–1^· dry* weight micromoles per minute per microgram of dry weight; *N^.^m* Newton metres; *pts* points; *s* seconds; *U/l* units per litre; *W* watts; *%* percentage

Symbols legends: ↓ indicates a within-group significant reduction. N.B. for case study, indicate a reduction from baseline; **↑** indicates a within-group significant improvement. N.B. for case study, indicate an increase from baseline; = indicates a within-group non-significant modification. N.B. for case study, indicate a similar value compared to baseline; * genetically assessed

Of note, due to heterogeneity in outcome reporting across studies, different reporting formats have been adopted in this table. Results are reported as mean ± SD unless otherwise specified.
